# On the origin of the widespread self-compatible allotetraploid *Capsella bursa-pastoris* (Brassicaceae)

**DOI:** 10.1038/s41437-021-00434-9

**Published:** 2021-04-19

**Authors:** Jörg A. Bachmann, Andrew Tedder, Marco Fracassetti, Kim A. Steige, Clément Lafon-Placette, Claudia Köhler, Tanja Slotte

**Affiliations:** 1 Department of Ecology, Environment and Plant Sciences, Science for Life Laboratory, Stockholm University, Stockholm, Sweden; 2 Department of Plant Biology, Swedish University of Agricultural Sciences & Linnean Center for Plant Biology, Uppsala, Sweden; 3 Present Address: School of Chemistry and Biosciences, Faculty of Life Sciences, University of Bradford, Bradford, UK; 4 Present Address: Institute of Botany, Biozentrum, University of Cologne, Cologne, Germany; 5 Present Address: Department of Botany, Faculty of Science, Charles University, Prague, Czech Republic

**Keywords:** Evolution, Polyploidy in plants, Plant evolution, Haplotypes

## Abstract

Polyploidy, or whole-genome duplication, is a common speciation mechanism in plants. An important barrier to polyploid establishment is a lack of compatible mates. Because self-compatibility alleviates this problem, it has long been hypothesized that there should be an association between polyploidy and self-compatibility (SC), but empirical support for this prediction is mixed. Here, we investigate whether the molecular makeup of the Brassicaceae self-incompatibility (SI) system, and specifically dominance relationships among *S-*haplotypes mediated by small RNAs, could facilitate loss of SI in allopolyploid crucifers. We focus on the allotetraploid species *Capsella bursa-pastoris*, which formed ~300 kya by hybridization and whole-genome duplication involving progenitors from the lineages of *Capsella orientalis* and *Capsella grandiflora*. We conduct targeted long-read sequencing to assemble and analyze eight full-length *S-*locus haplotypes, representing both homeologous subgenomes of *C. bursa-pastoris*. We further analyze small RNA (sRNA) sequencing data from flower buds to identify candidate dominance modifiers. We find that *C. orientalis*-derived *S-*haplotypes of *C. bursa-pastoris* harbor truncated versions of the male SI specificity gene *SCR* and express a conserved sRNA-based candidate dominance modifier with a target in the *C. grandiflora*-derived *S*-haplotype. These results suggest that pollen-level dominance may have facilitated loss of SI in *C. bursa-pastoris*. Finally, we demonstrate that spontaneous somatic tetraploidization after a wide cross between *C. orientalis* and *C. grandiflora* can result in production of self-compatible tetraploid offspring. We discuss the implications of this finding on the mode of formation of this widespread weed.

## Introduction

Whole-genome duplication, or polyploidization, is a common speciation mechanism in plants (Grant 1981). It has been estimated that about 15% of flowering plant speciation occurs through polyploidization (Wood et al. 2009), and most angiosperms have undergone whole-genome duplication at some point in their history (Masterson 1994; Soltis et al. 2004). Polyploidy has therefore had a marked impact on plant biodiversity. Understanding the origin and evolution of polyploids can thus give insights into an important process that generates biodiversity in flowering plants.

Polyploids share the common feature of harboring more than two complete chromosome complements, but differ in their mode of origin. Autopolyploids originate by duplication of the genome of one species, whereas allopolyploids originate by hybridization between two biological species, accompanied by genome duplication (Ramsey and Schemske 1998). Allopolyploidy can thus restore the fertility of otherwise sterile hybrids (Winge 1917). In general, both auto- and allopolyploids can form either through (1) the merger of unreduced (2*n*) gametes, (2) through a triploid bridge which involves the fusion of reduced (*n*) and unreduced (2*n*) gametes and subsequent crosses among triploids or backcrosses to diploids, or (3) through spontaneous somatic polyploidization involving meristematic tissue (Ramsey and Schemske 1998). Studies of polyploid formation have long focused on the role of unreduced gamete production, although instances of polyploid formation through spontaneous somatic polyploidization have been described (Ramsey and Schemske 1998). For instance, one of the first cases of allopolyploid formation described in detail occurred through somatic polyploidization in *Primula kewensis*, a hybrid between *Primula verticillata* and *Primula floribunda* (Newton and Pellew 1929).

Polyploidy can contribute to the rapid evolution of reproductive barriers and thus speciation, but newly formed polyploids face several challenges to establishment and persistence. A particular barrier to polyploid establishment is the lack of mates of the same ploidy. If the new polyploid is rare and mostly mates with the more frequent diploid parental species, most of its matings will be ineffective. This leads to selection against the rarer ploidy, a process termed minority cytotype exclusion (Levin 1975). This barrier can be alleviated in polyploids that are self-compatible and capable of self-fertilization or vegetative reproduction, or that are perennial (Grant 1956; Oswald and Nuismer 2011; Fowler and Levin 2016). If polyploids differ from their diploid progenitors in their ecological tolerance or show wider niche breadth, polyploid establishment should also be facilitated (Levin 1975, 1983; Fowler and Levin 2016).

An association between ploidy and mating system is expected on theoretical grounds, both because self-fertilization should facilitate polyploid establishment (Stebbins 1950, 1974) and because polyploids may experience less inbreeding depression than diploids (Lande and Schemske 1985; Ronfort 1999). In line with these expectations, Grant (1956) described a higher rate of self-fertilization in polyploid lineages relative to their diploid relatives, and Barrington (2007) documented an association between ploidy and self-fertilization rates in angiosperms, using phylogenetically independent contrasts. A corollary of the prediction of an association between mating system and polyploidy is that polyploidy should be associated with the breakdown of genetic self-incompatibility (SI) systems that allow rejection of self pollen in many outcrossing angiosperms. Therefore, polyploidy should be associated with self-compatibility (SC). In a broad comparative analysis, Mable (2004) found no global support for such an association across all plant families studied. However, in particular plant families with gametophytic SI, such as the Solanaceae, there is evidence for an association between polyploidy and SC (Mable 2004; Robertson et al. 2011).

One reason for the association between SC and polyploidy in Solanaceae species with gametophytic SI is that in this system breakdown of SI can be an instant byproduct of polyploidization (Zenil-Ferguson et al. 2019). This depends crucially on the molecular details of the Solanaceae SI mechanism, which is based on collaborative non-self recognition (Fujii et al. 2016). The female SI determinant consists of a style-expressed *S*-RNase which prevents pollen tube growth unless it is degraded by a pollen-expressed F-box protein. Each *S*-haplotype harbors multiple *S*-linked *F-box* genes that can detoxify several different maternal *S*-RNases, but not their own. In this system, the presence of two *S*-alleles in unreduced pollen grains can allow detoxification of any maternal *S*-RNase and therefore immediately leads to self-compatibility in polyploids (Kubo et al. 2010). As a result of the particular molecular basis of the Solanaceae SI system, a general association between polyploidy and self-compatibility is expected, and indeed observed (Zenil-Ferguson et al. 2019).

In contrast, in plant families such as the Brassicaceae, which has a sporophytic SI system that depends on self-recognition and rejection of self pollen, whole-genome duplication is not necessarily expected to cause the loss of SI. Nevertheless, there is evidence that dominance relationships among *S*-haplotypes in the Brassicaceae sporophytic SI system may facilitate the breakdown of SI in allopolyploids. In the Brassicaceae, it is common for only one allele of the male specificity determinant *SCR* to be expressed in heterozygotes, due to pollen-level dominance relationships among alleles. Such dominance relationships are mediated by sRNAs expressed by dominant alleles, that target and transcriptionally silence *SCR* of recessive alleles (Tarutani et al. 2010; Durand et al. 2014). In the presence of pollen level dominance, decay of key *S-*locus genes in the dominant *S-*haplotype would be sufficient for breakdown of SI in a newly formed allopolyploid (discussed in Tsuchimatsu et al. 2012). Decay of *S-*locus genes could involve silencing or nonfunctionalization of the female SI specificity determinant *SRK* or the male SI specificity determinant *SCR* in the dominant *S-*haplotype. If the dominant *S-*haplotype was already nonfunctional when the polyploid formed, then the recently formed allopolyploid could be instantly SC (Novikova et al. 2017).

In the Brassicaceae, there are empirical examples of SC allopolyploids where dominance seems to have played a role in the breakdown of SI. For instance, in the allopolyploid *Arabidopsis kamchatica*, breakdown of SI has been linked to decay of the male SI specificity gene *SCR* in a relatively dominant *S*-haplotype (Tsuchimatsu et al. 2012). The allopolyploid *Arabidopsis suecica* was likely instantly SC because it inherited a dominant nonfunctional *S*-allele harboring a 213-bp inversion in the *SCR* gene from its *A. thaliana* parent (Novikova et al. 2017). The dominant *A. thaliana-*derived *S-*haplotype of *A. suecica* is further likely to have suppressed the expression of *SCR* from *A. arenosa*, the SI parent of *A. suecica* (Novikova et al. 2017). The sRNA-based epigenetic mechanisms that regulate dominance of *S*-alleles at the pollen level in the Brassicaceae (Tarutani et al. 2010; Durand et al. 2014) could thus also facilitate breakdown of SI in Brassicaceae allopolyploids.

The allotetraploid weed Shepherd’s Purse *Capsella bursa-pastoris* (Brassicaceae) constitutes a suitable system in which to address the role of *S*-locus dominance for the loss of SI. *C. bursa-pastoris* is a self-fertilizing weedy species with a nearly worldwide distribution that is in part anthropogenic (Hurka and Neuffer 1997). The *Capsella* genus harbors three diploid species that differ in their mating system. The SC *C. orientalis* occurs in central Asia, the SC and self-fertilizing *C. rubella* is mainly found in the Mediterranean region and central Europe, and the SI outcrosser *C. grandiflora* is found mainly in the north-western Balkans and sometimes in northern Italy (Hurka et al. 2012). We have previously shown that *C. bursa-pastoris* is an allotetraploid species that originated ~200–300 kya through hybridization and genome doubling between an ancestor of the extant SC species *C. orientalis* and an SI progenitor ancestral to the extant species *C. grandiflora* and *C. rubella* (Douglas et al. 2015). This means that the ranges of the progenitor lineages must previously have been overlapping although currently they are not (Douglas et al. 2015). The two subgenomes of *C. bursa-pastoris* from the *C. grandiflora/rubella* lineage and the *C. orientalis* lineage are independently inherited, and have been designated the A and B subgenomes, respectively (Slotte et al. 2006, 2008; 2009; Douglas et al. 2015). It is further known that the maternal progenitor of *C. bursa-pastoris* came from the *C. orientalis* lineage (Hurka et al. 2012).

While we have a broad understanding of the genome composition and origin of *C. bursa-pastoris* (Douglas et al. 2015), we still know little about its mode of formation and the breakdown of SI in this species. Our analyses of *S-*locus variation so far suggest that *C. bursa-pastoris* could have been SC when it formed (Bachmann et al. 2019). For instance, the SC species *C. orientalis* harbors a dominant and nonfunctional *S-*haplotype with a coding frameshift mutation in the *SCR* gene, which is shared with *C. bursa-pastoris* B (Bachmann et al. 2019). This suggests that *C. orientalis* was SC when it gave rise to *C. bursa-pastoris*. If the *C. bursa-pastoris* A subgenome *S-*haplotype from the *C. grandiflora* lineage is more recessive than the B-haplotype from the *C. orientalis* lineage, then it is possible that the newly formed allotetraploid *C. bursa-pastoris* was immediately SC. Such instant SC might have facilitated establishment of the new allopolyploid.

In order to improve our understanding of the role of dominance at the *S-*locus for the origin of SC allopolyploid crucifers, we have conducted targeted long-read sequencing, annotation and analysis of eight full-length *S-*haplotypes representing both A and B subgenomes of *C. bursa-pastoris*. Using short-read data, we document low worldwide *S*-allele diversity at both subgenomes in a worldwide sample of *C. bura-pastori* accessions. We further analyze small RNA (sRNA) sequencing data from flower buds to investigate whether the *C. bursa-pastoris* B *S-*haplotype harbors and expresses sRNA-based dominance modifiers, which might have facilitated the shift to self-compatibility. Finally, we report on the spontaneous somatic tetraploidization and production of SC allotetraploid *Capsella* offspring after a wide cross between *C. orientalis* and *C. grandiflora*, which demonstrates that SC can be instant upon allopolyploid formation in this system. We discuss the implications of this finding on the mode of formation of *C. bursa-pastoris*.

## Materials and methods

### Plant material for *S*-locus and sRNA sequencing

We grew seeds from five accessions of *C. bursa-pastoris* from west and east Eurasia (Table S1) for production of bacterial artificial chromosome (BAC) libraries to be used for *S*-locus sequencing. These accessions were chosen to be representative of major geographic and genetic clusters in this species (Slotte et al. 2009; Cornille et al. 2016). Seed germination, plant growth and sampling of material for BAC library production followed the procedure described in Bachmann et al. (2018). Our material was sufficient for a total of four BAC libraries, one of which was based on two accessions (Table S1). For small RNA sequencing, we grew and collected mixed-stage flower buds from one *C. bursa-pastoris* accession (Tables S1 and S3) as described in Steige et al. (2016).

### *S*-locus sequencing and annotation

To obtain full-length *S-*haplotype sequences, we conducted targeted long-read sequencing of *S*-locus BACs, as described previously (Bachmann et al. 2018). Briefly, we extracted high molecular weight DNA from 10 g of flash-frozen young leaves per library to construct four *C. bursa-pastoris* BAC libraries (Table S1) at the French Plant Genomic Resource Centre CNRGV. We identified BACs with full-length *S*-haplotypes as in Goubet et al. (2012) by screening BAC libraries for *S*-locus flanking regions with DNA probes for *U-box* and *ARK3*, which flank the *S-*locus. We selected two different *S*-locus BACs per library, based on *S*-locus polymorphisms obtained by PCR and Sanger sequencing of *ARK3* and *U-box*. *S*-locus BACs were sequenced with long-read SMRT sequencing (Pacific Biosciences of California, CA, USA) to a coverage of 179–399 and short-read sequencing (MiSeq, Illumina, Inc., San Diego, USA) to a coverage of 1967–4380 at the National Genomics Infrastructure (NGI) in Uppsala, Sweden (Table S2). We generated eight *de novo S*-haplotype long-read assemblies, corresponding to the subgenome A and B *S*-haplotypes of the four *C. bursa-pastoris* BAC libraries. Assemblies were indel error corrected using short reads as described previously (Bachmann et al. 2018).

We annotated the *S*-locus sequences as previously described (Bachmann et al. 2018). In short, we used Maker v2.31.9 (Holt and Yandell 2011) running RepeatMasker v4.0.7 (http://www.repeatmasker.org) and Augustus v3.2.3 (Stanke et al. 2004) with *A. thaliana* as prediction species and protein sequences of ARK3, U-box and SRK for gene prediction. The highly divergent *SRK* was often not identified by Maker, which is why we identified *SRK* based on sequence similarity to known *SRK* sequences, as described in Bachmann et al. (2018). *SCR* was annotated by sequence similarity to *CoS12 SCR* (Bachmann et al. 2019) (subgenome B) with BLAST v2.5.0+ (Altschul et al. 1990), or an approach based on conservation of 8 cysteine residues in SCR (subgenome A), described in (Bachmann et al. 2018). For annotation of *SCR* in *C. bursa-pastoris* subgenome A we further used BLAST v2.5.0+ (Altschul et al. 1990) to annotate sequence similarities to two similar *S-*haplotypes, *A. lyrata SCR38* (Guo et al. 2011) and *C. rubella SCR* (Vekemans et al. 2014), using a minimum query (exon) coverage of 30 percent and e-value cutoff of 0.1.

To illustrate the phylogenetic relationship among *C. bursa-pastoris* A and B subgenome *S-*haplotypes and previously sequenced *S-*alleles, we constructed a phylogenetic tree based on an alignment of *SRK* exon 1 sequences from *C. bursa-pastoris* to a set of sequences downloaded from GenBank (Table S3) (Bachmann et al. 2018). Phylogenetic tree construction was done in RaXMl v8.2.3 (Stamatakis 2014) with the GTR + G model, and 100 bootstrap replicates. The tree was visualized using R v3.3 (R Core Team 2014). We further estimated nucleotide diversity and Watterson’s theta at *SRK* for both *S-*haplotypes based on our BAC sequences using Polymorphorama (Haddrill et al. 2008).

### *S*-locus coverage analyses based on whole-genome resequencing data of *C. bursa-pastoris*

To check whether a larger sample of *C. bursa-pastoris* might uncover additional *S-*haplotypes, we analyzed whole-genome resequencing data from 39 samples of *C. bursa-pastoris* sampled across the species’ distribution in Europe, east Eurasia and the Middle East (Table S4). Three of these sequences were newly generated, whereas the remainder were generated by previous studies (24 from Huang et al. (2018), 12 from Kryvokhyzha et al. (2019)). We also included whole-genome resequencing data from five *C. orientalis* individuals as a control (Table S4). Raw reads were trimmed with fastp (Chen et al. 2018) and mapped with bwa-mem (Li 2013) against a modified version of *C. rubella* genome (Slotte et al. 2013). In the modified reference, we masked the the *C. rubella S*-locus region (scaffold_7 7523601:7562919), and added the A and B alleles of the *S*-locus region of *C. bursa-pastoris* accession CbpWEDE, that we assembled previously. We selected only properly paired reads, calculated read coverage with samtools (Li et al. 2009) and visualized coverage across the A and B *S-*haplotypes for each accession using R v3.3 (R Core Team 2014).

### sRNA sequencing

To aid identification of expressed *S-*locus sRNA-based dominance modifiers, we extracted total RNA from mixed stage flower buds of *C. bursa-pastoris* accession CbpWEDE (Table S1) using the *mir*Vana extraction kit (Thermo Fisher Scientific Inc., Waltham MA, USA) which isolates RNA from 10-mer to kilobase length. Libraries for sequencing were prepared with the Illumina TruSeq small RNA kit (Illumina, Inc., San Diego, USA) at NGI Stockholm, Sweden. Samples were sequenced with a 1 × 51 setup and HiSeq Rapid SBS Kit v2 chemistry on a HiSeq2500 machine (Illumina, Inc., San Diego, USA). This yielded a total of 14 million reads for this accession.

### Identification of candidate dominance modifiers

We performed bioinformatic identification of sRNA precursor regions and targets in our *S-*locus BAC sequences, in combination with analyses of sRNA expression in flower buds to identify candidate dominance modifiers at the *C. bursa-pastoris S-*locus.

To identify putative sRNA precursor regions at the *S-*locus in our *S-*locus BACs, we followed the approach outlined in Durand et al. (2014) and Bachmann et al. (2019). First, we predicted inverted repeats in *C. bursa-pastoris S-*haplotypes using EMBOSS-einverted (Rice et al. 2000) with the following parameters: gap penalty = 8, match score = 4, mismatch score = 4, minimum score threshold = 50, maximum separation between start of repeat and end of inverted repeat = 350. We used an initial mapping of sRNA to screen for inverted repeats (Meyers et al. 2008; Durand et al. 2014), and checked the remaining inverted repeats for hairpin structure with *rnafold* (Lorenz et al. 2011) (Figs. S3 and S4) and kept hairpins with terminal loop smaller than 40 bp and a predicted hairpin structure >20 bp of high base-pairing probability with four or fewer mismatches and maximum two asymmetric bulges (Meyers et al. 2008; Durand et al. 2014).

To assess whether there was expression of sRNA in the precursor regions we identified, we analyzed our floral bud sRNA data. We used Trimmomatic v0.36 for removing sequencing adapters and for quality filtering of reads, before mapping small RNA of 18–27 nt length with STAR v2.5.3a (Dobin et al. 2013) to an adjusted genome of *C. rubella* (Slotte et al. 2013), where the *C. rubella S*-locus was replaced with a *C. bursa-pastoris* subgenome A or B *S-*locus, as in Bachmann et al. (2019), and the annotation of identified sRNA precursors predicted above. To identify targets of expressed candidate sRNA dominance modifiers, we used a modified Smith and Waterman algorithm (Smith and Waterman 1981) as in Durand et al. (2014) with the following scoring matrix: matches = +1; mismatches = −1; gaps = −2; G:U wobbles = −0.5 and a cutoff score for identifying targets of 18 (Burghgraeve et al. 2018).

### Spontaneous polyploidization in a wide cross of *C. orientalis* and *C. grandiflora*

In a previous study, we crossed *C. orientalis* and *C. grandiflora* for the purpose of generating a mapping population to investigate the genetic basis of loss of SI (Bachmann et al. 2019). In Bachmann et al. (2019) we presented analyses of the sequences of the *S-*haplotypes segregating in that cross, and identify a potential dominance modifier at the *C. orientalis S-*locus. Here, we report on the finding of spontaneous polyploidization in interspecific *C. orientalis* × *C. grandiflora* F1s and their F2 offspring, as these results are relevant to an improved understanding of potential pathways to formation of the allopolyploid *C. bursa-pastoris*. For the mapping of loss of SI in *C. orientalis*, tetraploid F2 offspring were not considered further in Bachmann et al. (2019).

To generate interspecific F1 hybrids, we crossed the maternal parent *C. orientalis* Co2008-1 with the paternal parent *C. grandiflora* Cg88.15 and viable F1 individuals were obtained via embryo rescue (Bachmann et al. 2019). A total of eight mature F1 were obtained, of which all where self-compatible and autonomously self-pollinating (Bachmann et al. 2019). We collected mature seeds from F1 plants in bulk from different flowering shoots. We measured the ploidy of 589 F2 individuals by estimating DNA content of fresh leaf tissue in relation to control samples of known ploidy and to *Carex acutiformis* using flow cytometry (Plant Cytometry Services, Didam, The Netherlands). We further measured ploidy on three six month old *C. orientalis* x *C. grandiflora* F1 plants (Partec Cube 6, Sysmex, Kobe, Japan).

### Self-compatibility of newly formed tetraploids

Due to the documented dominance of the *C. orientalis S-*haplotype over the *C. grandiflora S-*haplotype segregating in our F2s (Bachmann et al. 2019), we might expect instant self-compatibility in the tetraploid F2s. To assess whether this was the case, we scored 35 tetraploid F2s for silique elongation after autonomous self-pollination. Plants were scored as self-incompatible if they had no elongated siliques and otherwise as self-compatible. For a subset of four F2s we further scored pollen tube growth after manual self-pollination. To assess whether self-compatibility was stable across generations, we grew four F3 individuals that were offspring from a single self-compatible F2 plant and scored pollen tube growth after manual self-pollination. In these pollination assays we observed pollen tube elongation in the pistil after manual self-pollination of emasculated flowers, in six replicates per individual. We also used emasculated unpollinated pistils from one F2 and one F3 individual (in six replicates each) as negative controls. After 12 h flowers were fixed in a 9:1 solution of EtOH: glacial acetic acid for a minimum of 2 h. The flowers were then treated with 1 N NaOH at 60 °C for 20 min to soften the tissue and dipped into a staining solution with 0.01% decolorized aniline blue (in 2% K_3_PO_4_) for 2 h, before elongated pollen tubes in each pistil were observed under a Zeiss Axiovert epifluorescence microscope (Zeiss, Oberkochen, Germany). We tested for a difference in pollen tube counts between individuals and pollination treatments using a Kruskal–Wallis test in R 3.3.3.

## Results

### *S*-haplotype sequencing and annotation

We generated and annotated eight full-length *S-*haplotype sequences, four from the *C. bursa-pastoris* A (*C. grandiflora*-like) subgenome and four from the B (*C. orientalis*-like) subgenome. Final *S*-haplotype sequences (*U-box – ARK3*) were between 31,011 bp for subgenome A and 23,160–29,615 bp for subgenome B sequences (Table S2).

**Fig. 1:**
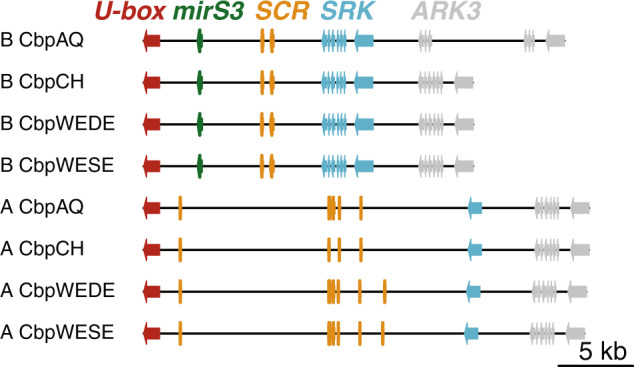
*Capsella bursa-pastoris* subgenome B and subgenome A *S-*haplotypes from four accessions. *S*-locus gene exons are shown as arrows in direction of transcription: *U-box* (red), *mirS3* (green), *SCR* (yellow), *SRK* (blue), and *ARK3* (gray). In *C. bursa-pastoris* subgenome A, we could not identify a complete *SCR* gene nor full-length *SCR* exons, and the bars for *SCR* here indicate short regions of similarity to *C. rubella SCR* and *A. lyrata AlySCR38*.

**Fig. 2:**
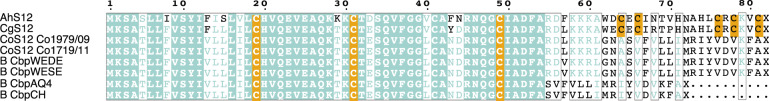
SCR amino acid alignment of *Arabidopsis halleri AhS12, Capsella grandiflora CgS12*, *C. orientalis CoS12* in accessions Co1979/09, Co1719/11 and *C. bursa-pastoris* subgenome B sequences. There is a single base-pair frame-shift in *C. orientalis* Co1979/09, Co1719/11 and in *C. bursa-pastoris* B CbpWEDE and B CbpWESE. There is a larger deletion in *C. bursa-pastoris* B CbpAQ and B CbpCH.

In the four *S-*haplotypes from the B subgenome, we identified the genes *ARK3, U-box* and *SRK* (Fig. 1). The male SI specificity gene *SCR* appears to be a pseudogene in all four *C. bursa-pastoris* B *S-*haplotypes. Two *C. bursa-pastoris* B *S-*haplotypes from western Eurasia shared a single-basepair coding frameshift deletion in *SCR* with *C. orientalis*, whereas two eastern Eurasian *C. bursa-pastoris* B *S-*haplotypes harbored a larger 31-bp frameshift coding deletion that overlapped with the single-basepair frameshift (Fig. 2). In the four *C. bursa-pastoris* A *S-*haplotypes, we identified the genes *ARK3, U-box*, and exon 1 of *SRK*. In addition we located short BLAST hits to the male SI specificity gene *SCR* (Fig. 1), but we were unable to identify the complete gene based on sequence similarity or using automated annotation procedures, including screening for a repetitive pattern of cysteine residues.

### Evolutionary genetic patterns at *SRK*

**Fig. 3:**
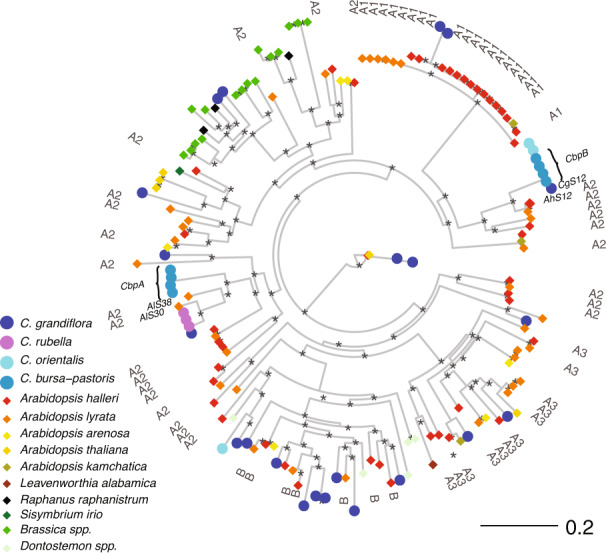
*SRK*-phylogeny. Maximum likelihood tree based on multiple sequence alignment of *SRK* exon 1 sequences. Large symbols indicate *Capsella* alleles. *Arabidopsis* alleles highly similar to *C. bursa-pastoris* subgenome B (*AlS38* and *AlS30*) and A (*AhS12*) and *SRK* are annotated in the figure. Nodes with >95% bootstrap support are marked with an asterisk (*). The tree is rooted using *ARK3* sequences. Labels in the outer circle indicate dominance classes according to Mable et al. (2018) (A1 < B < A2, A3 in order of increasing dominance).

*SRK* exon 1 sequences from *C. bursa-pastoris* B were phylogenetically close to those from *C. orientalis*, as expected, whereas those from *C. bursa-pastoris* A were closest to sequences *S38* and *S30* from *A. lyrata*, *C. rubella* sequences and *C. grandiflora CgS37* (Fig. 3). All subgenome A *S-*haplotype sequences clustered together, separate from subgenome B *S-*haplotype sequences (Fig. 3). Both subgenomes harbored very low levels of diversity at *SRK* in our geographically broad sample (coding region diversity was π = 0.0009, θ_W_ = 0.0009 for *SRK* B and π = 0.0018, θ_W_ = 0.0014 for *SRK* A).

### Coverage analyses of 39 accessions support low *S*-allele diversity at the *C. bursa-pastorisS*-locus

To investigate whether a broader sample of *C. bursa-pastoris* accessions would support our inference of limited diversity of *S-*haplotypes at each of the two subgenomes, we analyzed publicly available whole-genome resequencing data from 39 *C. bursa-pastoris* accessions sampled worldwide. By mapping short reads to a modified *C. rubella* reference genome supplemented with BAC sequences of the A and B *S-*haplotypes of *C. bursa-pastoris* accession CbpWEDE, we identified accessions that shared these haplotypes as those that show broad and even coverage across each *S-*haplotype. We first demonstrated that mapping short reads of *C. orientalis* to such a reference, broad and even coverage was retreived only for the B *S-*haplotype (Figs. S1 and S2, Table S4). We then inspected coverage plots for *C. bursa-pastoris*. Overall, all 39 *C. bursa-pastoris* accessions showed evidence of harboring the same *S-*haplogroup at the A subgenome, and at the B subgenome all accessions except one accession from Kryvokhyzha et al. (2019) showed broad and even coverage, indicating that they harbor the same *S*-haplotype (Figs. S1 and S2, Table S4). These results thus support our conclusion that *C. bursa-pastoris* harbors limited *S-*allele diversity within each of its two subgenomes.

### Candidate dominance modifiers and targets

**Fig. 4:**
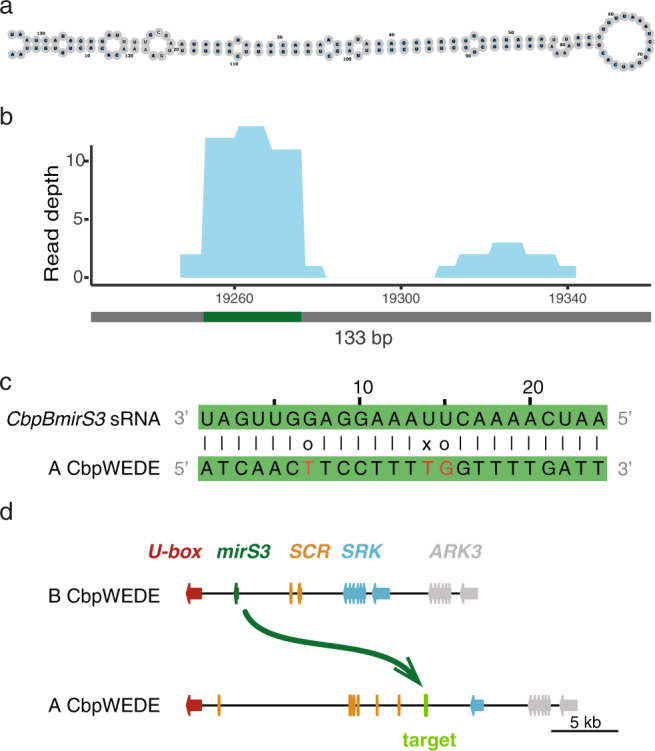
sRNA precursor *CbpBmirS3* identified in the B subgenome of accession CbpWEDE. **a** Predicted hairpin structure of *CbpBmirS3* with *rnafold*. **b** Expression of *CbpBmirS3* in *C. bursa-pastoris* subgenome B *S-*haplotype. The gray bar marks the length of the precursor, and the green bar shows the location of expressed 24 nt sRNA with a target predicted in subgenome A. **c** Predicted target of *CbpBmirS3* expressed 24 nt sRNA in subgenome A. **d** Schematic of predicted dominance with *C. bursa-pastoris* subgenome B expressing *CbpBmirS3* sRNA that target *C. bursa-pastoris* subgenome A.

We identified a total of 50 inverted repeats in *C. bursa-pastoris* subgenome B, out of which three were retained as putative dominance modifiers based on sRNA expression data (Table S5, Fig. S3). In *C. bursa-pastoris* B, one of the precursor regions, here termed *CbpBmirS3* (Fig. 4a), is homologous to *ComirS3*. *ComirS3* is a putative sRNA-based dominance modifier that we previously identified as associated with dominance of SC in *C. orientalis* (Bachmann et al. 2019) and that shows sequence conservation with *AhMirS3* in *A. halleri* (Durand et al. 2014). Small RNAs are expressed in flower buds from *CbpBmirS3* in *C. bursa-pastoris* accession CbpWEDE (Fig. 4b). We found one potential target region of *CbpBmirS3* sRNA in the *C. grandiflora* derived subgenome A *S*-locus of *C. bursa-pastoris* with an affinity >18 (Durand et al. 2014; Burghgraeve et al. 2018), within 2 kb of the closest BLAST hit of *SCR* (Fig. 4d, e). One putative dominance modifier in *C. bursa-pastoris* subgenome A was retained from precursor prediction (Table S5, Fig. S4), with no target of expressed sRNA predicted in *C. bursa-pastoris* subgenome B (Durand et al. 2014; Burghgraeve et al. 2018). The combination of sequence conservation, small RNA expression and the existence of a target in the *C. bursa-pastoris* A *S-*haplotype together make *CbpBmirS3* a promising candidate dominance modifier, although difficulties in annotating *SCR* in A subgenome sequences prevent us from directly assessing its impact on *SCR* expression.

### Spontaneous polyploidization in a wide cross of *C. orientalis* and *C. grandiflora*

For the purpose of mapping the genetic basis of loss of SI in *C. orientalis*, we generated F2 offspring from a cross between *C. orientalis* and *C. grandiflora*. After a routine ploidy screen we unexpectedly found a substantial proportion of tetraploid F2 individuals (~18%, *n* = 105 tetraploids out of 589 screened F2s). We then screened the ploidy of three *C. orientalis* × *C. grandiflora* F1 hybrid individuals and found that one of them harbored both tetraploid and diploid flowering shoots. These results suggest a potential pathway to allopolyploid formation in *Capsella* through wide hybridization followed by spontaneous polyploidization.

### Self-compatibility of newly formed tetraploids

**Fig. 5:**
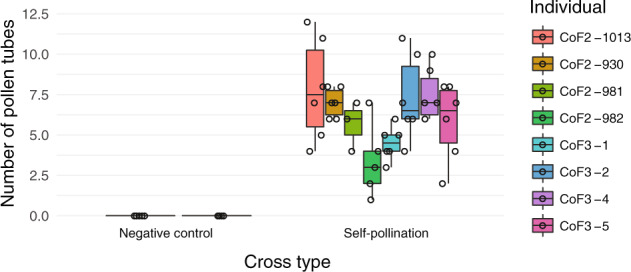
Pollen tube counts in newly formed tetraploids with and without manual self-pollination. Boxplots show the number of pollen tubes after manual self-pollination of emasculated flowers of tetraploid F2 individuals and F3 offspring of tetraploid F2s and in negative controls consisting of unpollinated emasculated tetraploid F2 and F3 flowers.

Due to the documented dominance of the *C. orientalis S-*haplotype over the *C. grandiflora S-*haplotype segregating in our F2s (the diploid F1 was self-compatible; Bachmann et al. 2019), we expected that tetraploid F2s might be instantly self-compatible. In agreement with this expectation, all 35 tetraploid F2s had elongated siliques and were scored as self-compatible, although there was variation in the number of elongated siliques per plant. Our pollination assays revealed a significant difference in pollen tube counts among individuals and pollination treatments, with self-pollinated tetraploid F2s and F3s having higher pollen tube counts than negative controls (Fig. 5; Kruskal–Wallis rank-sum test statistic = 33.018, *P* = 0.0001, df = 9), as expected if assayed tetraploid F2s and F3s were self-compatible. This result therefore demonstrates that instant self-compatibility is a possible outcome upon allopolyploid formation in the Brassicaceae.

## Discussion

Here, we have generated and analyzed eight full-length *S-*haplotypes representing both homeologous subgenomes of the allotetraploid crucifer *C. bursa-pastoris*, with the aim to investigate the role of dominant *S-*haplotypes for the transition to self-compatibility. More specifically, we were interested in examining whether the same sRNA-based dominance modifiers that are important for pollen-level dominance (Durand et al. 2014) might also facilitate transitions to self-compatibility in allopolyploid crucifers.

In line with a previous study (Bachmann et al. 2019) we found that all *C. bursa-pastoris* B *S-*haplotypes clustered together and harbored *C. orientalis-*derived mutations that are expected to result in the expression of a truncated and likely nonfunctional SCR protein. We further found that all our *C. bursa-pastoris* samples harbored very similar A *S*-haplogroup sequences, and polymorphism was very low at *SRK* of both subgenomes (θ_W_ = 0.0009 for *SRK* B; θ_W_ = 0.0014 for *SRK* A). *S-*locus coverage analyses based on whole-genome resequencing data from 39 *C. bursa-pastoris* accessions sampled worldwide further support our inference that the diversity of *S-*haplotypes is very low at both the *C. bursa-pastoris* A and B subgenomes. While low polymorphism at the B *S-*haplogroup might be expected given the limited diversity in the parental species *C. orientalis* (Douglas et al. 2015), limited polymorphism at the *C. bursa-pastoris* A *S-*haplogroup is more surprising given the high number of *S-*haplotypes currently segregating in *C. grandiflora* (Guo et al. 2009). While our results suggest that *C. bursa-pastoris* only inherited one *S-*haplotype from each of its parental species, a single polyploid origin of *C. bursa-pastoris* from two haploid gametes has previously been ruled out based on the presence of shared polymorphisms across the genome between *C. bursa-pastoris* and both of its parental species (Douglas et al. 2015). Other possible explanations for the low *S-*diversity at the A subgenome of *C. bursa-pastoris* include bottlenecks associated with the origin of *C. bursa-pastoris* or selection favoring a specific A subgenome *S-*haplotype after allopolyploid formation.

If the *C. bursa-pastoris* B *S-*haplotype was dominant over the A *S-*haplotype derived from the paternal *C. grandiflora/C. rubella* lineage, then the transition to SC might have occurred immediately upon allopolyploid formation. Therefore, we investigated whether the B *S-*haplotype harbored candidate sRNA-based dominance modifiers, which are known to confer pollen-level dominance in SI Brassicaceae (Tarutani et al. 2010; Durand et al. 2014). Using a combination of bioinformatic analyses and analyses of newly generated floral bud sRNA data, we identified an *S-*linked candidate dominance modifier termed *CbpBmirS3* that is encoded by the *C. orientalis-*derived B subgenome, and that is expressed in flower buds. The *CbpBmirS3* candidate dominance modifier has a predicted target in the A *S-*haplotype of *C. bursa-pastoris*. We further identified one potential dominance modifier in the *C. grandiflora-*derived A *S-*haplotype, however there were no targets of expressed sRNAs in the *C. bursa-pastoris* B *S-*haplotype. Taken together, these results suggest that dominant suppression of a recessive *SCR* allele derived from the *C. grandiflora/rubella* lineage by dominance modifiers expressed by the *C. orientalis-*derived dominant *S-*haplotype could have contributed to the transition to SC in the newly formed allotetraploid *C. bursa-pastoris*. These results therefore mirror those recently published regarding the transition to SC in the allotetraploid *A. suecica*, where a dominant loss-of-function allele at the *A. thaliana-*derived *S-*locus is thought to have conferred instant SC (Novikova et al. 2017).

A limitation of our study is that we were unable to empirically verify that *SCR* from the A *S-*haplotype is indeed repressed in *C. bursa-pastoris*, because we could not confidently annotate *SCR* in the A *S-*haplotype. We could further only annotate the first exon of the *SRK* gene in the A *S-*haplotype. The extremely polymorphic genes *SCR* and *SRK* are known to be notoriously difficult to annotate (e.g., Guo et al. 2009 and Vekemans et al. 2014). Therefore, it is possible that we were unable to fully annotate these genes due to bioinformatic limitations. Another possibility is that mutations have degraded *C. bursa-pastoris* A *SCR* and *SRK* to the point that they are no longer recognizable. At present we cannot distinguish between these scenarios. However, we note that *CbpBmirS3* exhibits sequence conservation to the *C. orientalis* candidate dominance modifier *ComirS3*, which has been shown to be associated with dominant suppression of expression of *SCR* of more recessive *S-*haplotypes in diploid *S-*heterozygotes (Bachmann et al. 2019), although we have not directly assessed its effect in the tetraploid individuals studied here. Furthermore, pollination assays in spontaneously generated *C. orientalis* × *C. grandiflora* tetraploids support our hypothesis that instant self-compatibility is possible upon hybridization and polyploidization in the Brassicaceae. However, the *C. grandiflora S-*haplotype segregating in our F2 individuals likely belongs to a different *S-*haplogroup, with a different position in the dominance hierarchy among *S-*alleles than that of *C. bursa-pastoris* A (Fig. 3). Specifically, the most closely related *SRK* sequences to *C. bursa-pastoris* A belong to the dominance class A2, which is a more dominant allele class than the *C. grandiflora* allele segregating in our F2s, which likely belongs to the most recessive A1 class, according to the classification of Mable et al. (2018). This is important because whether instant self-compatibility upon allopolyploidization is expected depends on the dominance relationships among *S-*haplotypes inherited from the parental species of a newly formed allopolyploid.

In this study we report on the spontaneous generation of tetraploid offspring after a wide cross between *C. orientalis* and *C. grandiflora*, the two parental lineages of *C. bursa-pastoris*. Due to the direct observation of somatic tetraploidization in the F1 generation and the high proportion of tetraploid F2 offspring produced, we propose that tetraploid offspring likely formed through seed production by tetraploid flowering branches on F1 individuals. Based on this finding we propose that spontaneous somatic tetraploidization following wide hybridization might be a plausible pathway to formation of the widespread weed *C. bursa-pastoris*, as in the classic case of *Primula kewensis* (Newton and Pellew 1929). While unreduced gametes are currently considered to be the main route to auto- and allopolyploid formation (Ramsey and Schemske 1998; Mason and Pires 2015; Kreiner et al. 2017a; 2017b), our findings underscore the need for further systematic assessment of the contribution of somatic doubling.

Here, we have investigated the potential mode of formation and transition to SC of the allotetraploid species *C. bursa-pastoris*. Our results suggest that sRNA-based dominance modifiers may have been important, and we propose a potential mode of formation through spontaneous polyploidization after hybridization, possibly accompanied by instant self-compatibility. The high-quality full-length *S-*haplotype sequences presented here provide a basis for future detailed investigation of genetic variation and selection at the *S-*locus in this widespread allopolyploid species.

## Supplementary information

Supplementary Information

## Data Availability

*S*-locus sequences, sRNA sequences and whole-genome resequencing data have been uploaded to ENA (https://www.ebi.ac.uk/ena) under project accession numbers PRJEB38903, PRJEB32122 and PRJEB41157.
