# Supplementary material for: On the origin of the widespread self-compatible allotetraploid Capsella bursa-pastoris (Brassicaceae)
Source: Heredity (Edinb). 2021 Apr 19;127(1):124–34. doi: 10.1038/s41437-021-00434-9 (PMC8249383; doi:10.1038/s41437-021-00434-9)
Supplement: Supplementary file 1 — Supplementary Information [file 41437_2021_434_MOESM1_ESM.pdf]

Supplementary Information

Table S1. Overview of plant accessions used for BAC and sRNA sequencing.

**Species**	**Accession (abbreviation)**	**Populati on**	**Origin**[1]	**ENA study accession number**	**Purpose**
C. bursa-pastoris	CbpWEDE9.136 (CbpWEDE)	WEDE9	DE	LR596595 (B) PRJEB38903 (A)	*S*-locus BAC sequencing sRNA sequencing
C. bursa-pastoris	CbpWESE11.139 (CbpWESE)	WESE11	SE	LR596610 (B) PRJEB38903 (A)	*S*-locus BAC sequencing
C. bursa-pastoris	CbpKMB205 (CbpCH)	KMB	CH	LR596597[2] (B) PRJEB38903 (A)	*S*-locus BAC sequencing
C. bursa-pastoris	CbpGY36 (CbpCH)	GY	CH	LR596597[2] (B) PRJEB38903 (A)	*S*-locus BAC sequencing
C. bursa-pastoris	CbpAQ416 (CbpAQ)	AQ	CH	LR596611[2] (B) PRJEB38903 (A)	*S*-locus BAC sequencing

[1]Geographical origin; CH=China, DE=Germany, SE=Sweden. More information on the origin of the *C. bursa-pastoris* samples is available in (Slotte *et al.* 2009).
[2]One BAC library containing material from both accessions was produced.

Table S2. Information on sequence length and coverage for SMRT and MiSeq sequencing of BAC clones.

**Species**	**Accession**	**Coverage SMRT raw assembly (x)**	**SMRT N50 read length (bp)**	**MiSeq coverage (x)**	**Final length of *S*-locus contig**
C. bursa-pastoris B	CbpWEDE9.136	246	24 893	4 215	114 127
C. bursa-pastoris B	CbpWESE11.139	399	25 568	4 380	99 499
C. bursa-pastoris B	CbpAQ416	338	25 653	3 157	131 978
C. bursa-pastoris B	CbpCH (CbpKMB205, CbpGY36)[1]	252	27 925	1 967	189 744
C. bursa-pastoris A	CbpWEDE9.136	224	25 110	2 681	131 935
C. bursa-pastoris A	CbpWESE11.139	230	25 224	2 703	150 295
C. bursa-pastoris A	CbpAQ416	179	26 453	2 769	127 194
C. bursa-pastoris A	CbpCH (CbpKMB205, CbpGY36)[1]	185	25 631	2 694	146 951

[1] One BAC library containing material from both accessions CbpKMB205 and CbpGY36 was produced, and the resulting *S*-haplotype sequence is designated CbpCH1 her

Table S3. *SRK* accessions used for phylogenetic reconstruction in this study

Species	Accession						
Arabidopsis arenosa	JX464654.1	JX464627.1	JX464615.1	JX464623.1	JX464618.1	JX464616.1	
Arabidopsis halleri	KJ772390.1	KJ772396.1	GQ915357.1	GQ915356.1	GQ915355.1	GQ915360.1	GQ915354.1	GQ915358.1
	GQ915353.1	GQ915351.1	GQ915352.1	GQ915359.1	EU075137.1	GQ915363.1	GQ915361.1	KJ461471.1
	KJ461478.1	KJ461479.1	KJ461484.1	EU878014.1	EU878015.1	EU878012.1	EU075125.1	EU075136.1
	EU075138.1	EU075143.1	EU075131.1	EU075132.1	EU075133.1	EU075134.1	GQ915364.1	GQ915365.1
	EU075140.1	EU878009.1	EU075130.1	EU075124.1	EU075135.1	EU878011.1	EU878010.1	EU075127.1
	EU075142.1							
Arabidopsis kamchatica	JX114764.1	JX114771.1	JX114756.1					
Arabidopsis lyrata	GQ915366.1	GQ915359.1	HQ379629.1	KJ772405.1	KJ772408.1	KJ772418.1	HQ379630.1	HQ379631.1
	AF328993.1	AF328999.2	AF328996.2	AF328997.2	AF328990.2	AF328992.2	AF328994.2	GQ915369.1
	GQ915370.1	AF329000.2	AF328995.1	AF328998.2	FJ867321.1	AF328991.2	JX464650.1	JX464654.1
	EU878023.1	EU878024.1	EU878025.1	JX464639.1	EU878016.1	EU878019.1	EU878021.1	EU878020.1
	JX464644.1	EU878017.1						
Arabidopsis thaliana	AY772640.1	AY772641.1	AY772644.1	GQ915361.1				

Brassica napus	AB086976.1	AJ245479.1	AY448031.1	AY448033.1	AY448035.1			
Brassica oleracea	AJ306587.1	JX861859.1	GQ915361.1	AB070624.1	AJ306591.1	AJ306584.1		
Brassica rapa	AB070625.1	AY448029.1	AY448025.1	AY448027.1				
Capsella grandiflora	DQ530638.1	DQ530642.1	DQ530637.1	FJ613333.1	DQ530640.1	DQ530639.1	FJ613332.1	FJ613331.1
	DQ530641.1	FJ649961.1	FJ649962.1	FJ649959.1	FJ649957.1	FJ649956.1	FJ649955.1	FJ649953.1
	FJ613330.1	FJ649960.1	FJ649958.1					
Capsella rubella	FJ649926.1	FJ649937.1	FJ649942.1					
Dontostemon integrifolius	HE687285.1							
Dontostemon micranthus	HE687283.1	HE687282.1						
Dontostemon senilis	HE687287.1	HE687284.1						
Leavenworthia alabamica	JQ714260.1							
Raphanus raphanistrum	KP117083.1	KP117085.1	KP117084.1					
Sisymbrium irio	JX114771.1							

Table S4. *Capsella* accessions with short-read data mapped to the allele S-locus alleles A and B. The sample id column indicates the species and accession, with Cbp prefix for *C. bursa-pastoris* samples and Co prefix for *C. orientalis* samples. The DP_tot column contains information on median of read depth across the genome, DP_A and DP_B contains information on median read depth across S-locus alleles A and B, respectively. The study accession number, sampling location and original reference is also given. In these analyses, *C. orientalis* was used as a control, as it should harbor an *S*-allele matching the B *S*-allele but not the A *S*-allele from *C. bursa-pastoris*.

**Sample id**	**DP_tot**	**DP_A**	**DP_B**	**Accession number**	**Reference**	**Location**
Cbp_FR50	11	6	5	SRR6382382	Kryvokhyzha et al. 2019	North Europe
Cbp_SE33	18	9	8	SRR6382393	Kryvokhyzha et al. 2019	North Europe
Cbp_STA4	20	7	8	SRR6382400	Kryvokhyzha et al. 2019	North Europe
Cbp_STJ2	15	7	7	SRR6382399	Kryvokhyzha et al. 2019	North Europe
Cbp_POG15	56	31	30	ERS5293359	this study	South Europe
Cbp_POG18	66	41	36	ERS5293360	this study	South Europe
Cbp_POG21	66	41	39	ERS5293361	this study	South Europe
Cbp_AL87	14	6	5	SRR6382386	Kryvokhyzha et al. 2019	Middle East
Cbp_JO56	32	13	14	SRR6382384	Kryvokhyzha et al. 2019	Middle East
Cbp_TR73	21	7	7	SRR6382397	Kryvokhyzha et al. 2019	Middle East
Cbp_JO59	43	25	24	SRR8904466	Kryvokhyzha et al. 2019	Middle East
Cbp_DUB-RUS9	56	34	0	SRR8904462	Kryvokhyzha et al. 2019	Central Asia
Cbp_KYRG-3-14	28	17	16	SRR8904463	Kryvokhyzha et al. 2019	Central Asia
Cbp_LAB-RUS-4	21	13	7	SRR8904464	Kryvokhyzha et al. 2019	Central Asia
Cbp_TACH-CHIN14	54	35	31	SRR8904465	Kryvokhyzha et al. 2019	Central Asia
Cbp_BEJ3	20	14	12	SRR6179229	Huang et al. 2018	North West China
Cbp_FY5	21	14	12	SRR6179230	Huang et al. 2018	North West China
Cbp_TACH1	29	20	18	SRR6179231	Huang et al. 2018	North West China
Cbp_HRB135	23	14	14	SRR6179232	Huang et al. 2018	East China
Cbp_HRB138	21	13	13	SRR6179233	Huang et al. 2018	East China
Cbp_QD325	19	13	12	SRR6179234	Huang et al. 2018	East China
Cbp_HD63	23	14	13	SRR6179235	Huang et al. 2018	East China
Cbp_HF257	24	14	15	SRR6179236	Huang et al. 2018	East China
Cbp_NJN7	22	15	14	SRR6179237	Huang et al. 2018	East China
Cbp_WH54	23	15	14	SRR6179238	Huang et al. 2018	East China
Cbp_SH7	22	14	14	SRR6179239	Huang et al. 2018	East China
Cbp_AQ415	22	14	13	SRR6179241	Huang et al. 2018	East China
Cbp_FJ7	23	14	13	SRR6179252	Huang et al. 2018	East China
Cbp_NE2	23	13	13	SRR6179243	Huang et al. 2018	East China
Cbp_JJN2	20	12	12	SRR6179244	Huang et al. 2018	East China
Cbp_LL761	22	14	13	SRR6179245	Huang et al. 2018	West China
Cbp_ZD31	21	13	12	SRR6179246	Huang et al. 2018	West China
Cbp_LJH9	21	14	13	SRR6179247	Huang et al. 2018	West China
Cbp_LJ5	22	13	13	SRR6179248	Huang et al. 2018	West China
Cbp_ZD13	20	13	12	SRR6179249	Huang et al. 2018	West China
Cbp_ZJ15	21	13	13	SRR6179250	Huang et al. 2018	West China
Cbp_GY6	23	15	14	SRR6179251	Huang et al. 2018	West China
Cbp_YNHZ2	21	13	13	SRR6179253	Huang et al. 2018	West China
Cbp_HuY3	22	13	12	SRR6179242	Huang et al. 2018	West China
Co_GUB-RUS5	35	0	40	SRR8904471	Kryvokhyzha et al. 2019	Central Asia
Co_PAR-RUS	31	0	35	SRR8904459	Kryvokhyzha et al. 2019	Central Asia
Co_QH-CHIN4	29	0	32	SRR8904460	Kryvokhyzha et al. 2019	Central Asia
Co_URAL-RUS4	29	0	32	SRR8904461	Kryvokhyzha et al. 2019	Central Asia
Co_FY1	21	0	24	SRR6179226	Huang et al. 2018	West China

Table S5. sRNA precursor prediction in *C. bursa-pastoris* haplotypes

**Species**	**Accession**	**Subgenome**	***S*-locus length**	**Predicted inverted repeats with einverted**	**sRNA precursors (expressed, hairpin)**	**sRNA precursors with predicted sRNA targets in other subgenome (Score ≥18)**
C. bursa-pastoris	CbpWEDE9.136	B	23198	50	3	1 (*CbpBmirS3*)
C. bursa-pastoris	CbpWEDE9.136	A	31155	76	1	0

Supplementary Figures

Figure S1. Plots of sequence coverage across the *C. bursa-pastoris* A *S*-locus, for 39 *C. bursa-pastoris* accessions sampled worldwide, and five *C. orientalis* accessions (as a control). The y-axis shows coverage relative to median genome-wide coverage, with likely repetitive regions with elevated coverage (higher than twofold the genome-wide median) shaded in grey. The x-axis shows the position in bp on the *C. bursa-pastoris* A WEDE *S*-haplotype. Coloured boxes above the coverage plots indicate the position of exons of the genes *U-box* (red), *SCR* (yellow), *SRK* (blue) and *ARK3* (grey).

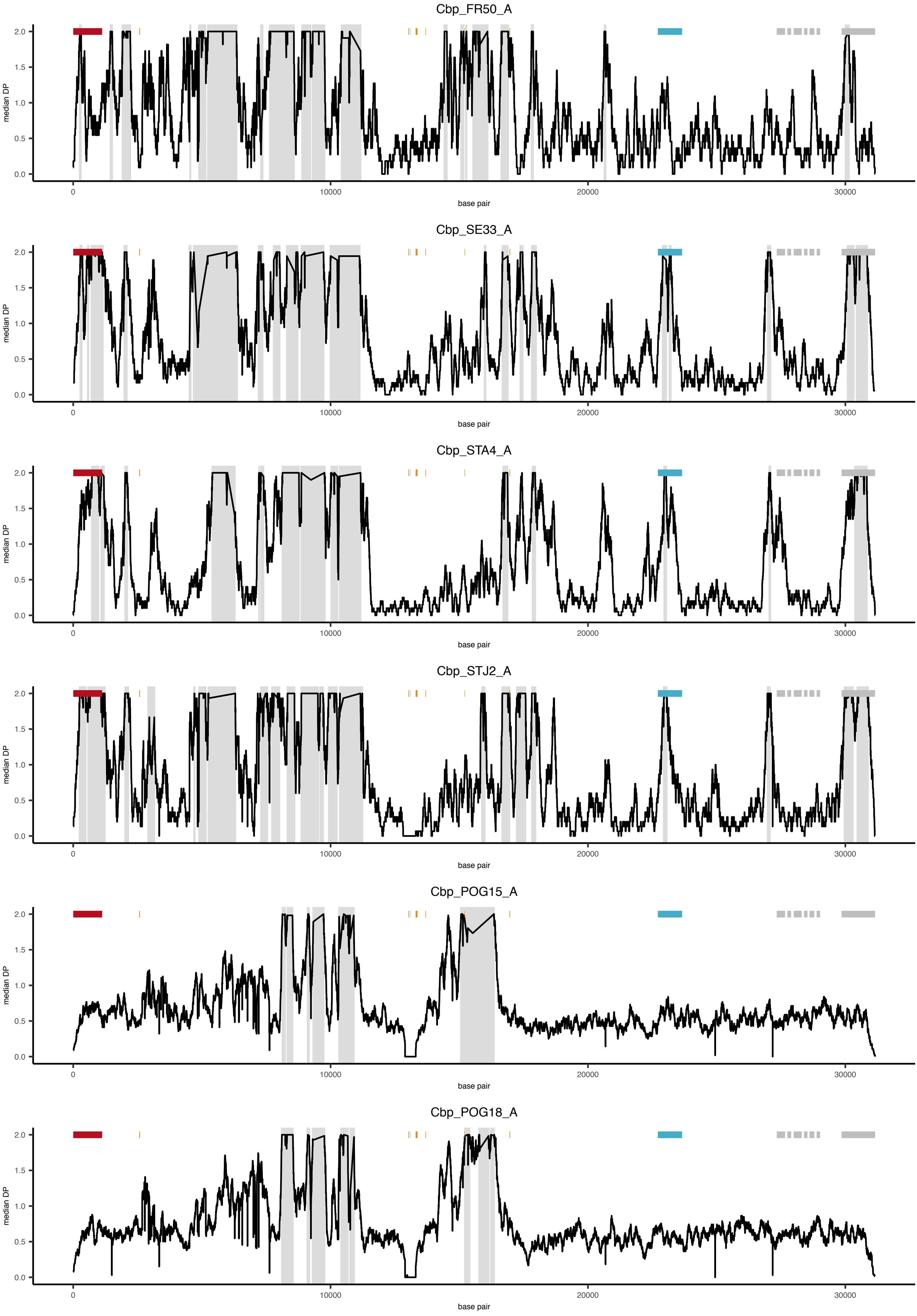
Cbp_FR50_A
Cbp_SE33_A
Cbp_STA4_A
Cbp_STJ2_A
Cbp_POG15_A
Cbp_POG18_A
median DP
base pair
0.0
0.5
1.0
1.5
2.0
0
10000
20000
30000

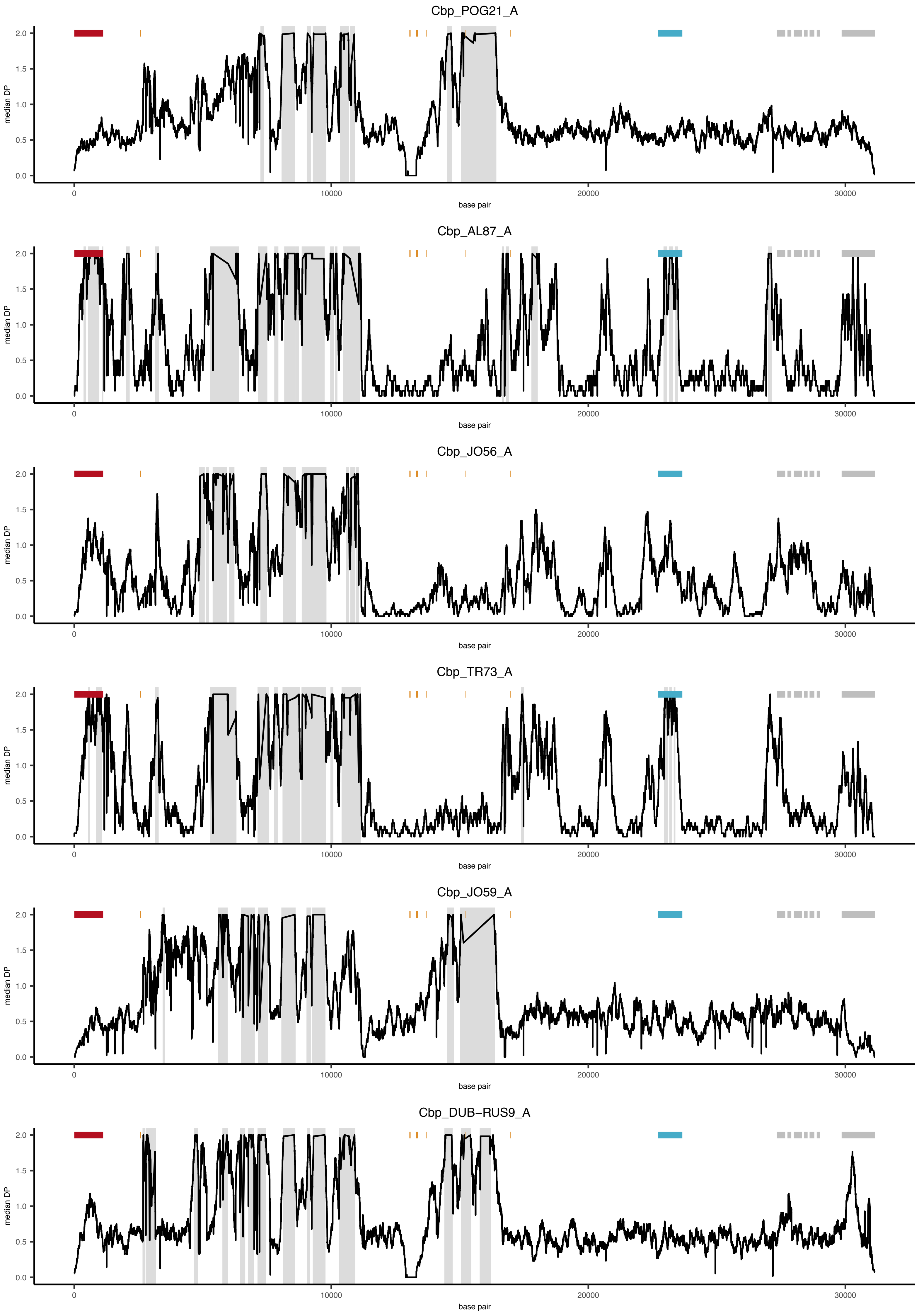
Cbp_POG21_A
Cbp_AL87_A
Cbp_JO56_A
Cbp_TR73_A
Cbp_JO59_A
Cbp_DUB−RUS9_A
median DP
base pair
0.0
0.5
1.0
1.5
2.0
0
10000
20000
30000

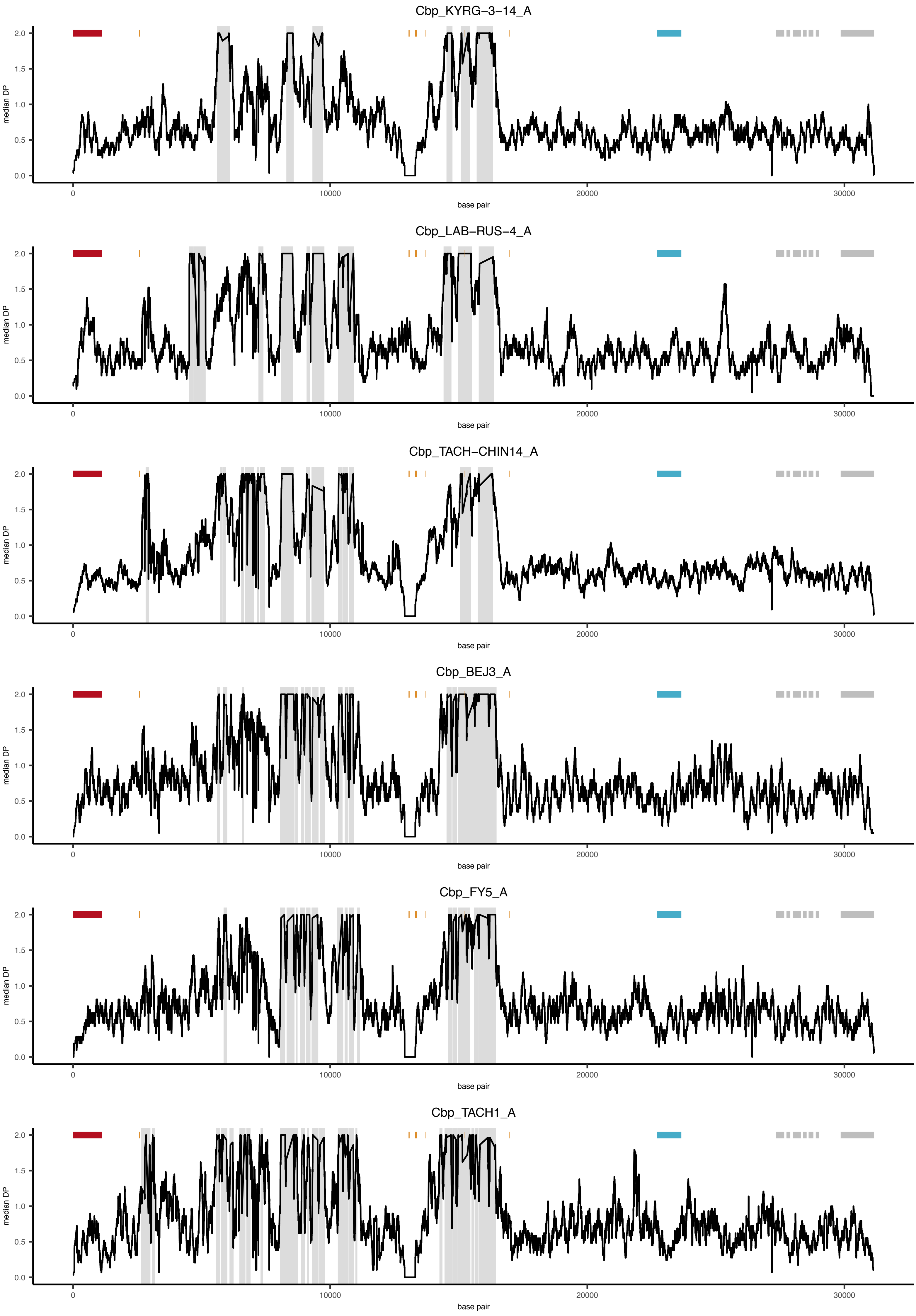

Cbp_KYRG−3−14_A
Cbp_LAB−RUS−4_A
Cbp_TACH−CHIN14_A
Cbp_BEJ3_A
Cbp_FY5_A
Cbp_TACH1_A
median DP
base pair
0
10000
20000
30000
0.0
0.5
1.0
1.5
2.0

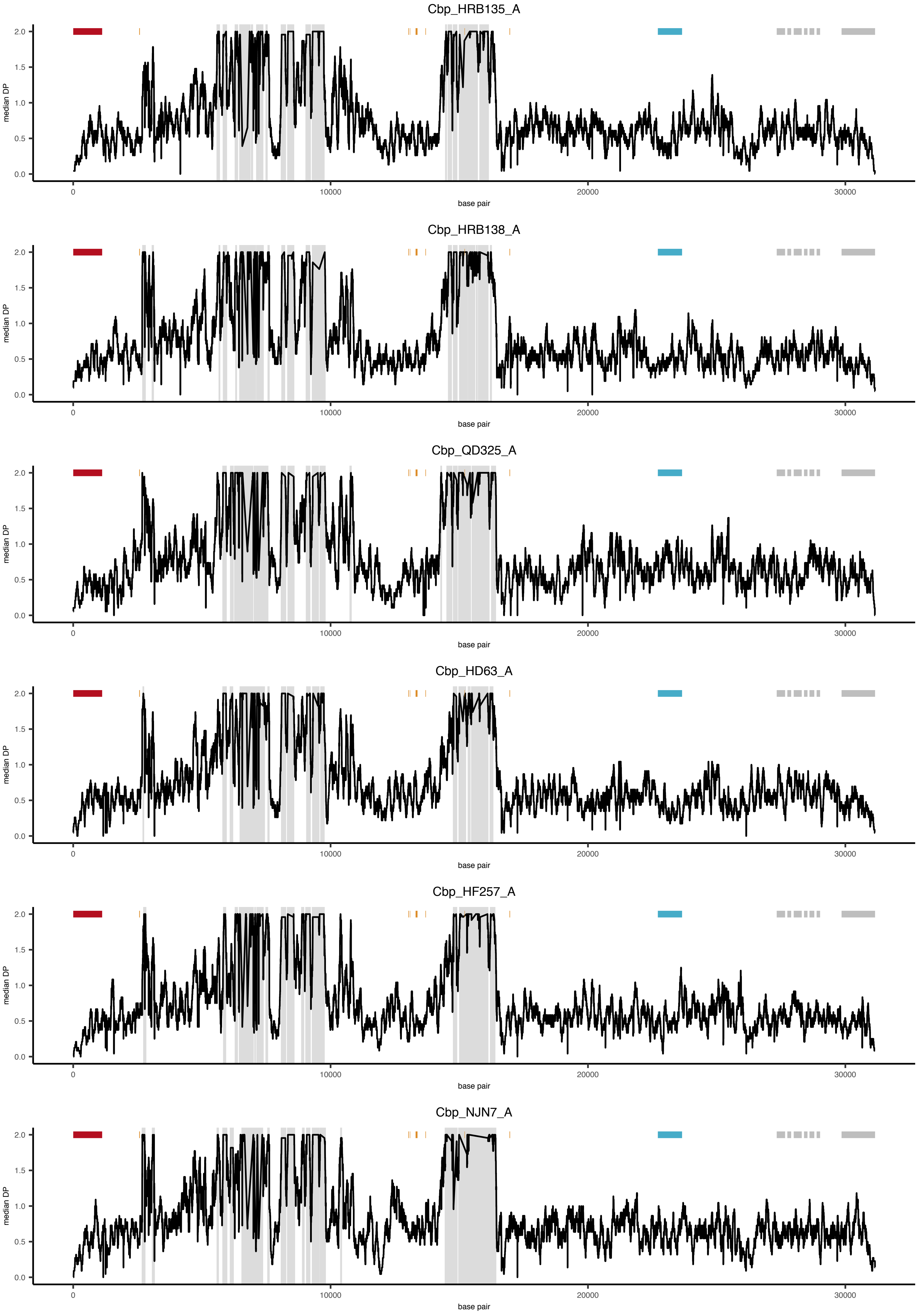
Cbp_HRB135_A
Cbp_HRB138_A
Cbp_QD325_A
Cbp_HD63_A
Cbp_HF257_A
Cbp_NJN7_A
median DP
base pair
0.0
0.5
1.0
1.5
2.0
0
10000
20000
30000

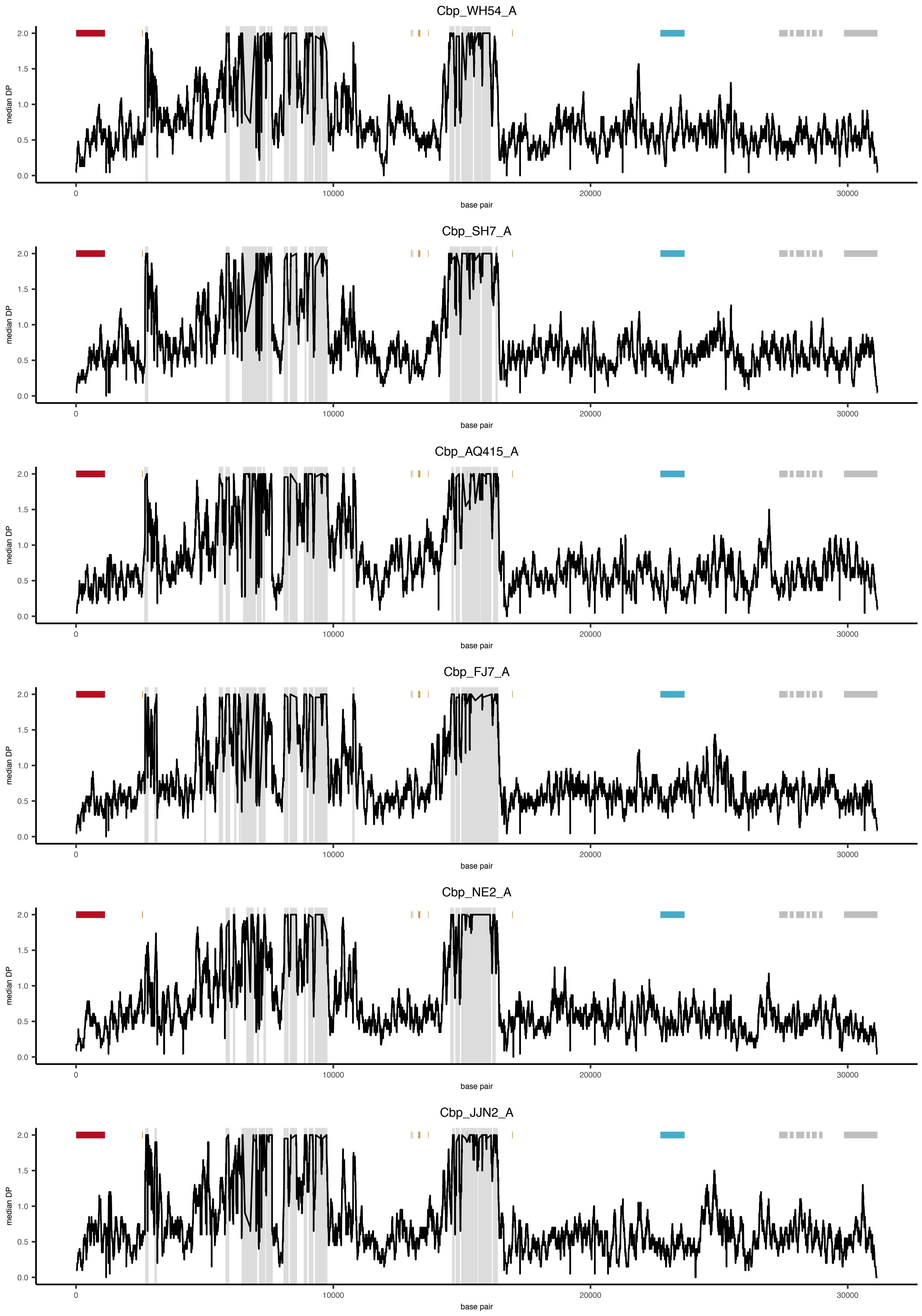
Cbp_WH54_A
Cbp_SH7_A
Cbp_AQ415_A
Cbp_FJ7_A
Cbp_NE2_A
Cbp_JJN2_A
median DP
base pair
0.0
0.5
1.0
1.5
2.0
0
10000
20000
30000

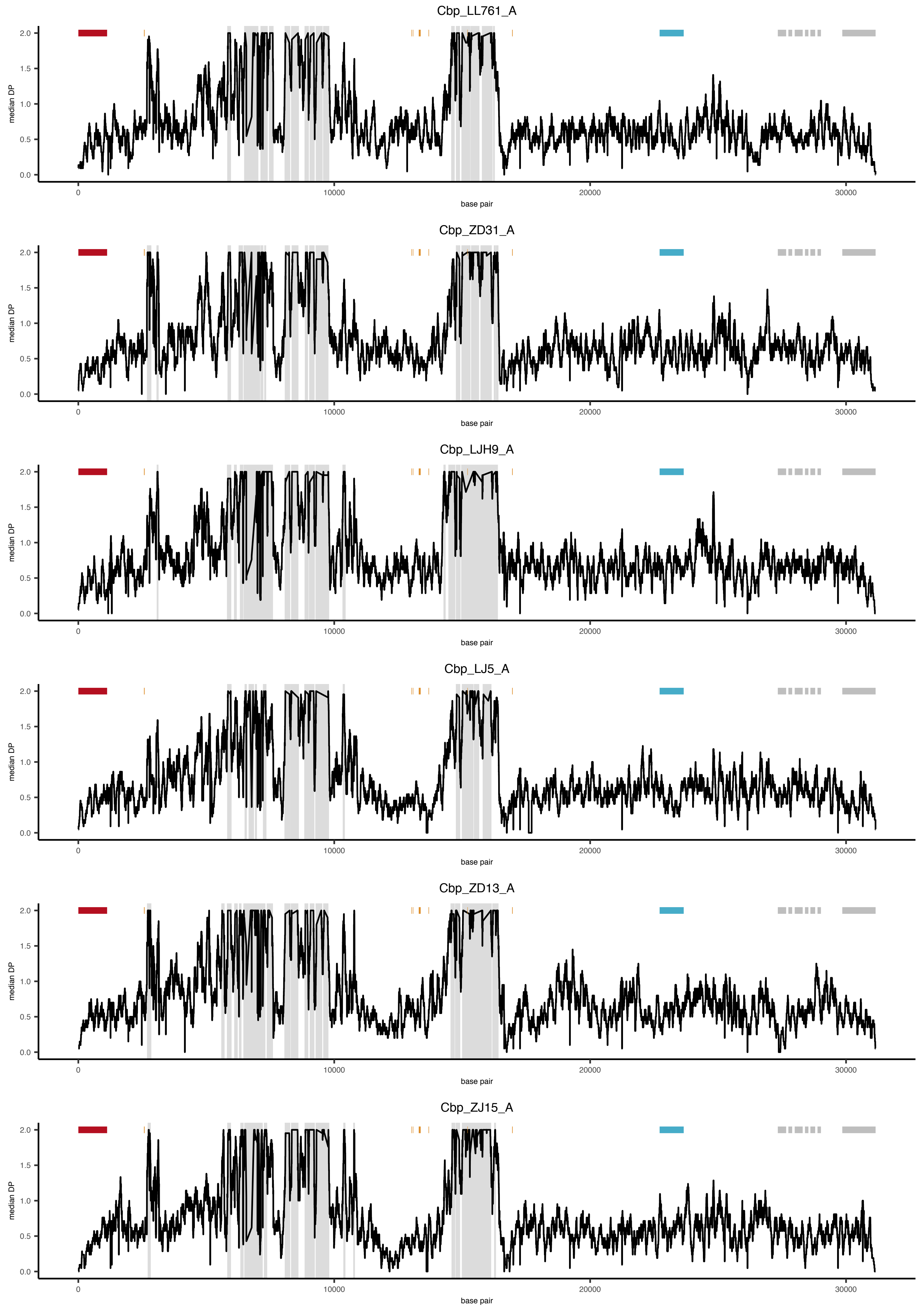

Cbp_LL761_A
Cbp_ZD31_A
Cbp_LJH9_A
Cbp_LJ5_A
Cbp_ZD13_A
Cbp_ZJ15_A
median DP
base pair
0.0
0.5
1.0
1.5
2.0
0
10000
20000
30000

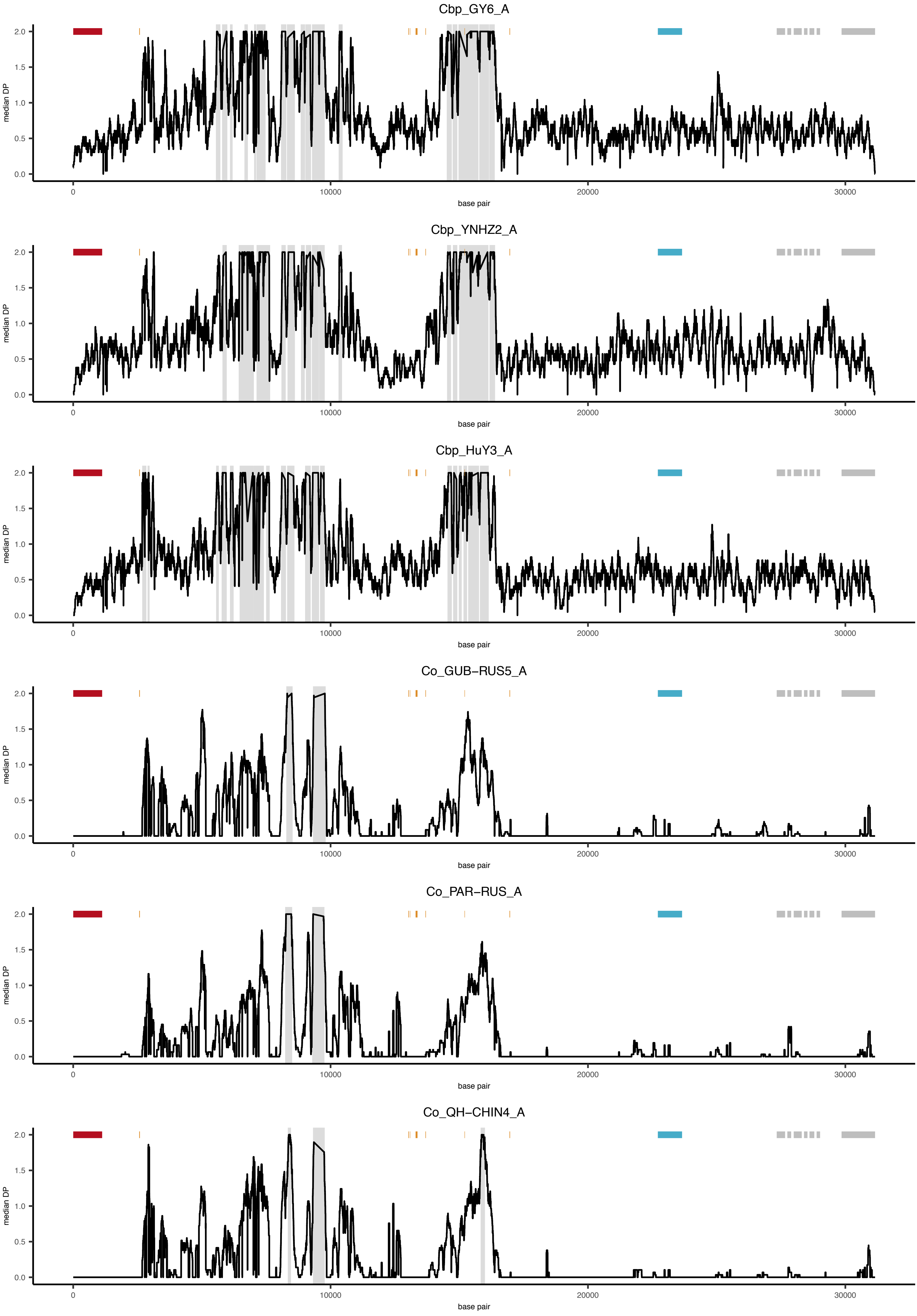

Cbp_GY6_A
Cbp_YNHZ2_A
Cbp_HuY3_A
Co_GUB−RUS5_A
Co_PAR−RUS_A
Co_QH−CHIN4_A
median DP
base pair
0.0
0.5
1.0
1.5
2.0
0
10000
20000
30000

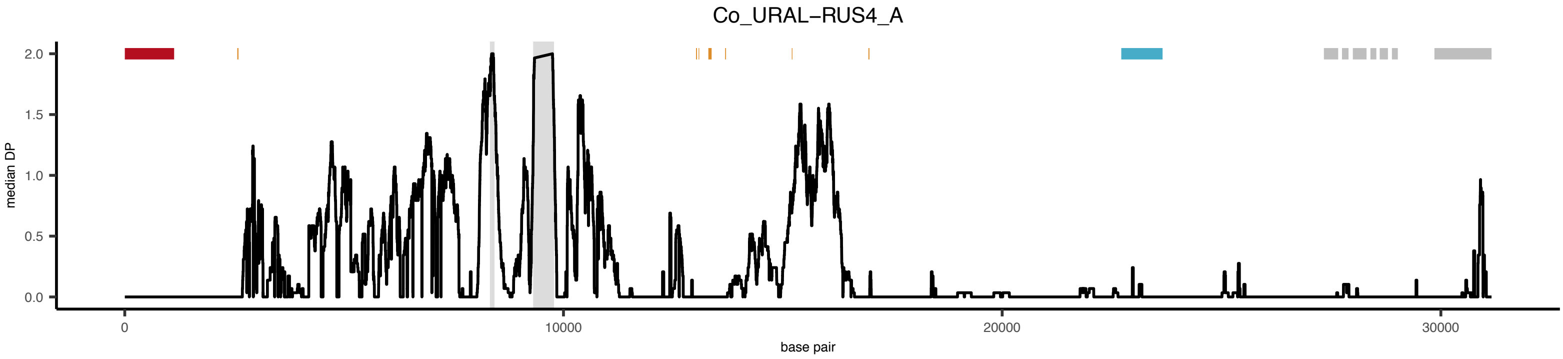
Co_URAL−RUS4_A
median DP
2.0
1.5
1.0
0.5
0.0
0
10000
20000
30000
base pair

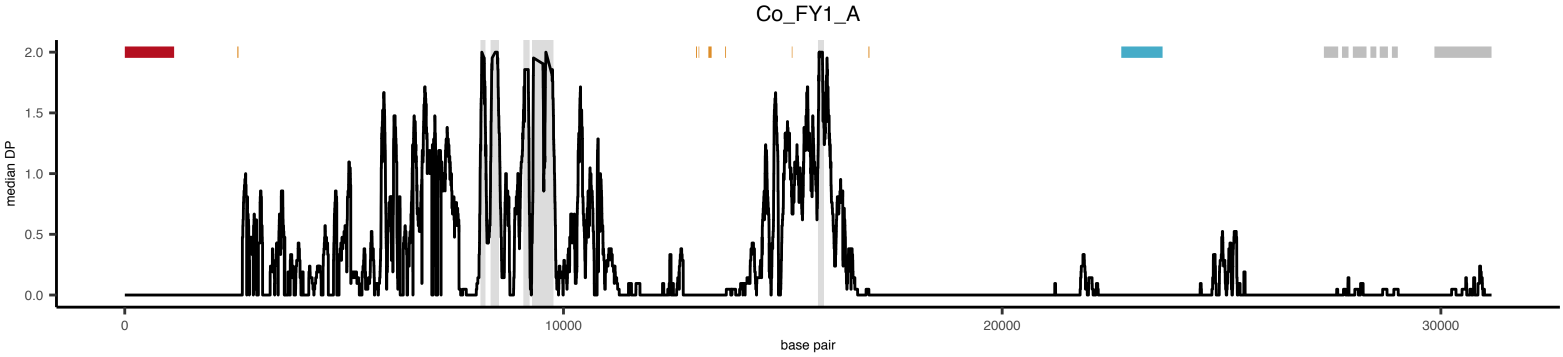
Co_FY1_A
median DP
2.0
1.5
1.0
0.5
0.0
0
10000
20000
30000
base pair

Figure S2. Plots of sequence coverage across the *C. bursa-pastoris* B *S*-locus, for 39 *C. bursa-pastoris* accessions sampled worldwide, and five *C. orientalis* accessions (as a control). The y-axis shows coverage relative to median genome-wide coverage, with likely repetitive regions with elevated coverage (higher than twofold the genome-wide median) shaded in grey. The x-axis shows the position in bp on the *C. bursa-pastoris* B WEDE *S*-haplotype. Coloured boxes above the coverage plots indicate the position of exons of the genes *U-box* (red), *mirS3* (green), *SCR* (yellow), *SRK* (blue) and *ARK3* (grey).

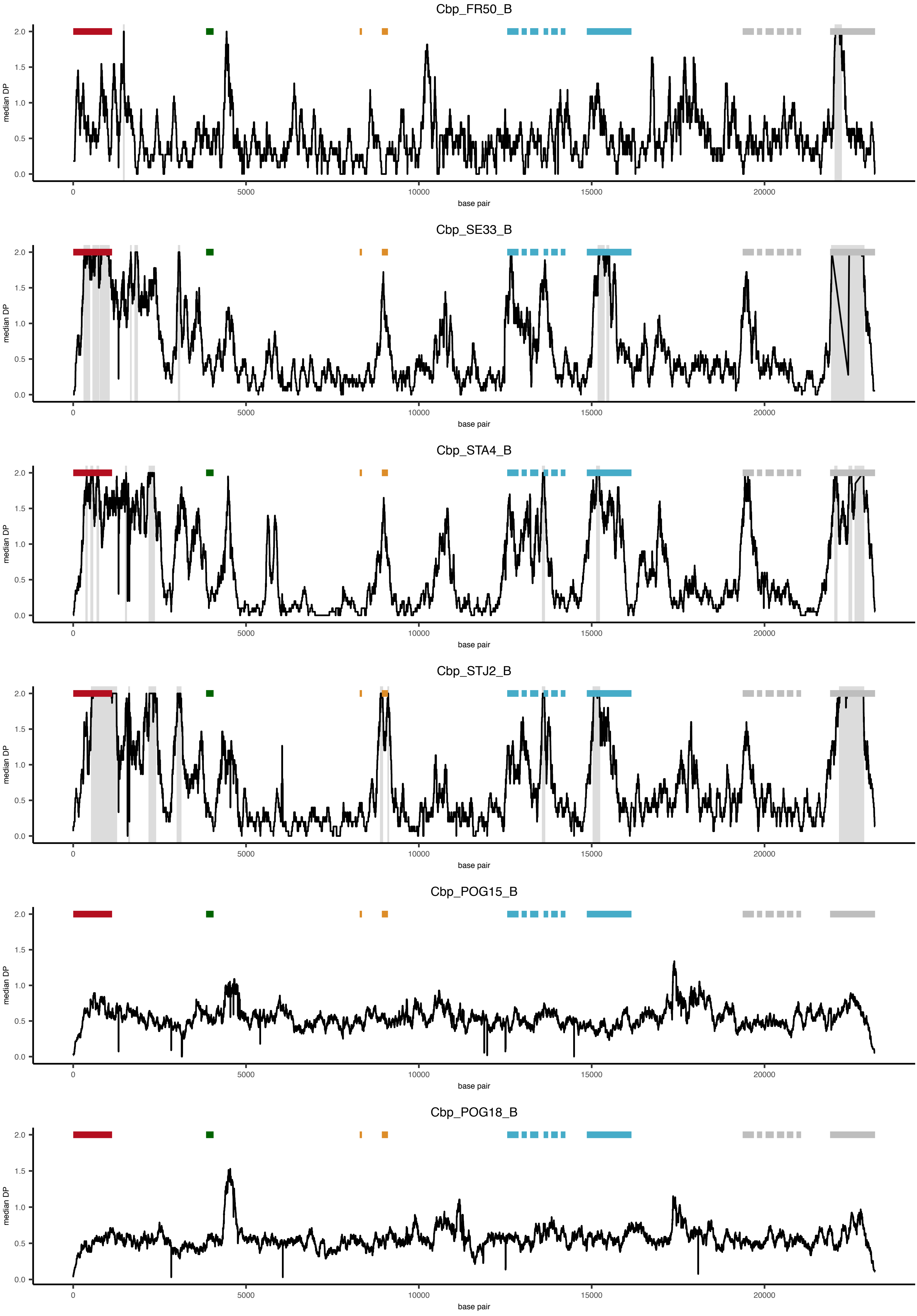

Cbp_FR50_B
Cbp_SE33_B
Cbp_STA4_B
Cbp_STJ2_B
Cbp_POG15_B
Cbp_POG18_B
median DP
base pair
0.0
0.5
1.0
1.5
2.0
0
5000
10000
15000
20000

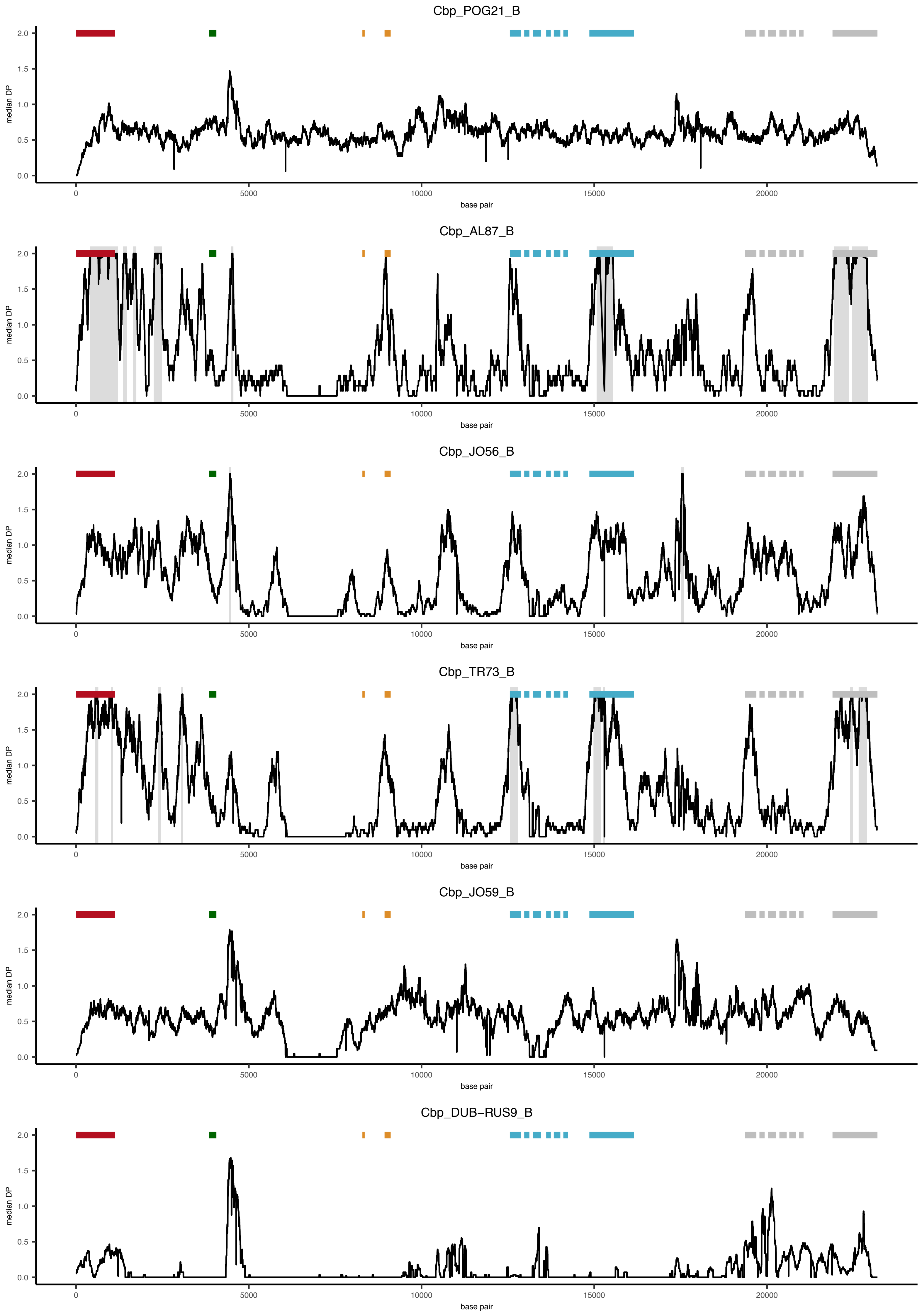
Cbp_POG21_B
Cbp_AL87_B
Cbp_JO56_B
Cbp_TR73_B
Cbp_JO59_B
Cbp_DUB−RUS9_B
median DP
base pair
0.0
0.5
1.0
1.5
2.0
0
5000
10000
15000
20000

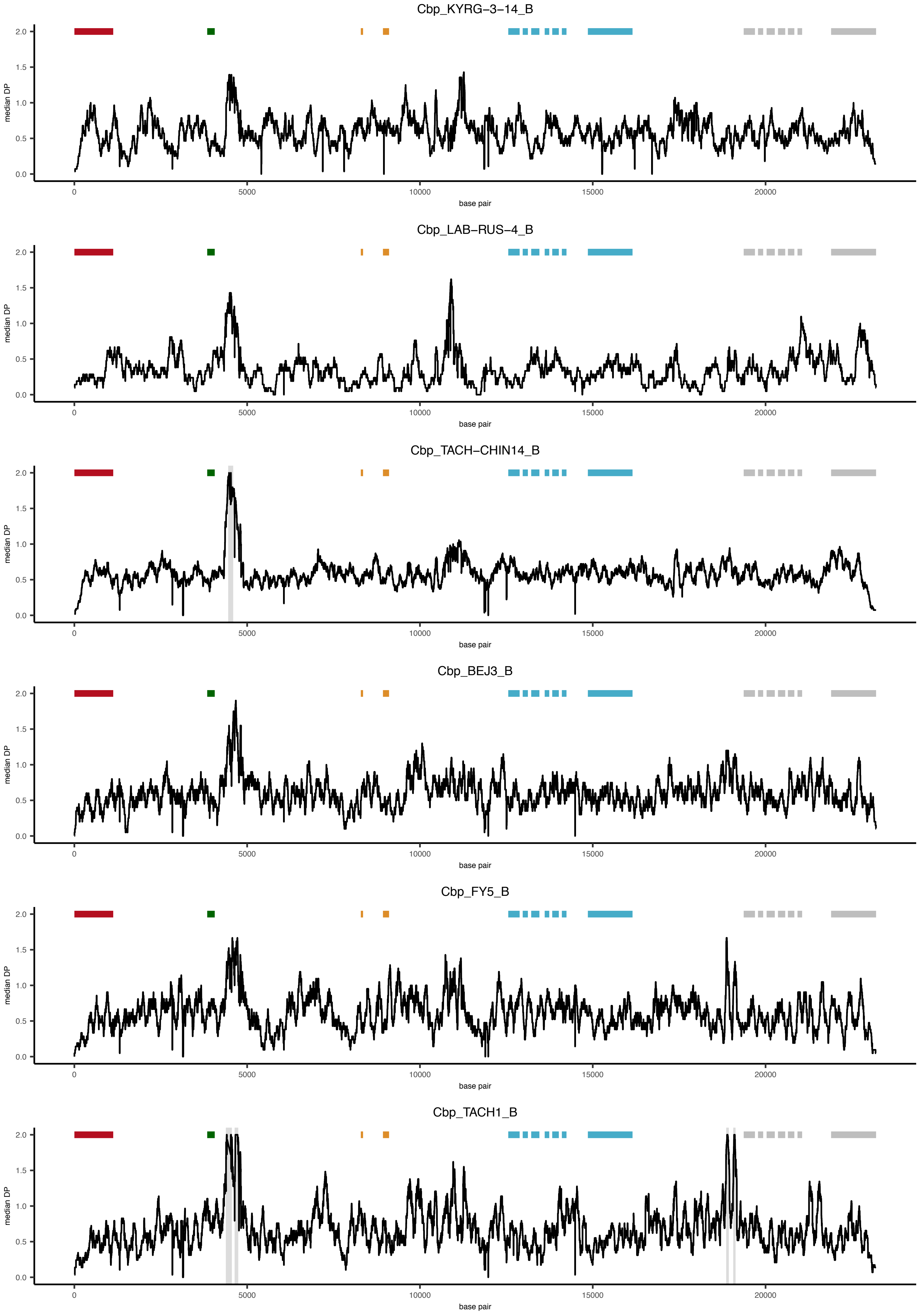

Cbp_KYRG-3-14_B
Cbp_LAB-RUS-4_B
Cbp_TACH-CHIN14_B
Cbp_BEJ3_B
Cbp_FY5_B
Cbp_TACH1_B
median DP
base pair
0.0
0.5
1.0
1.5
2.0
0
5000
10000
15000
20000

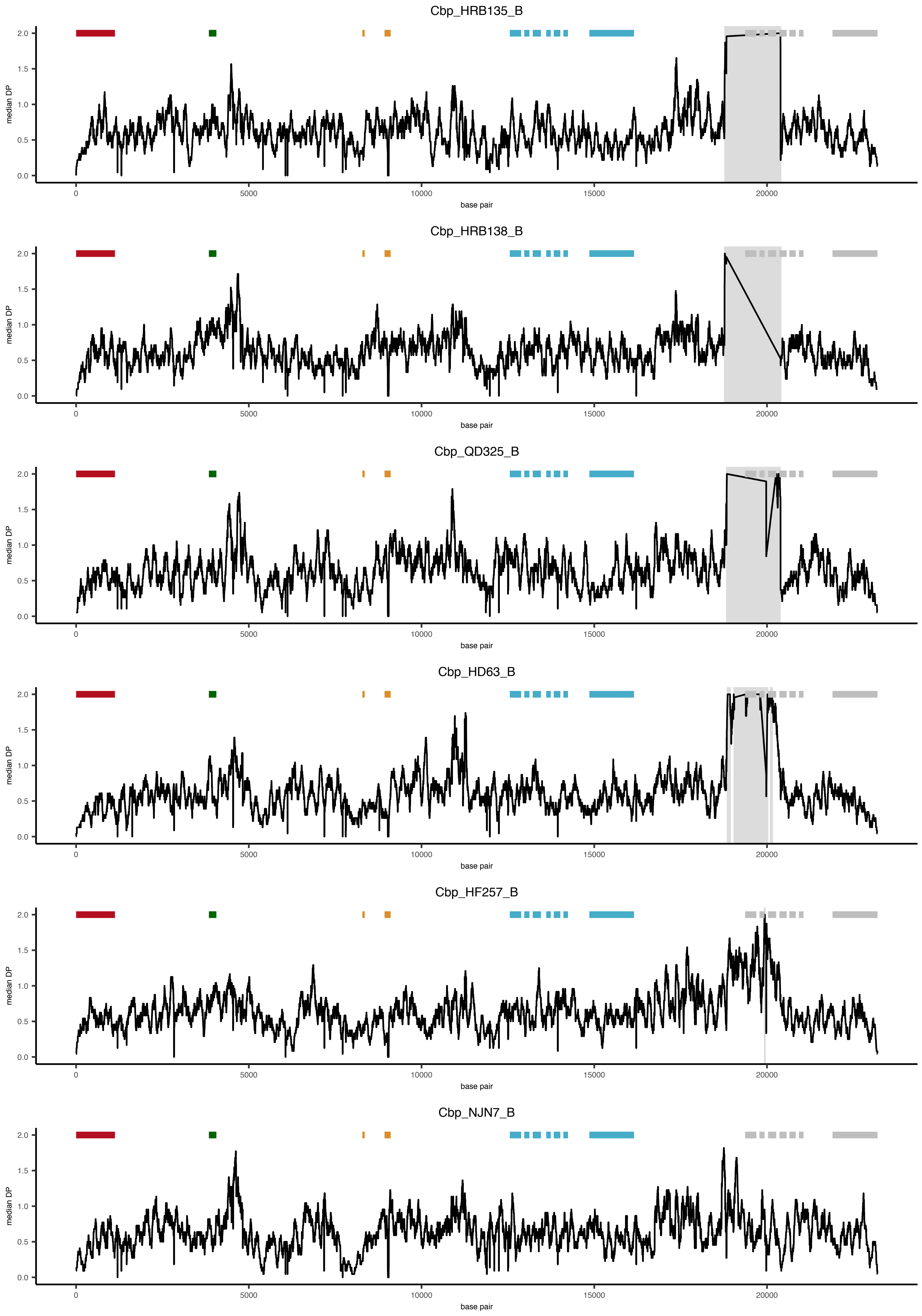
Cbp_HRB135_B
Cbp_HRB138_B
Cbp_QD325_B
Cbp_HD63_B
Cbp_HF257_B
Cbp_NJN7_B
median DP
base pair
0.0
0.5
1.0
1.5
2.0
0
5000
10000
15000
20000

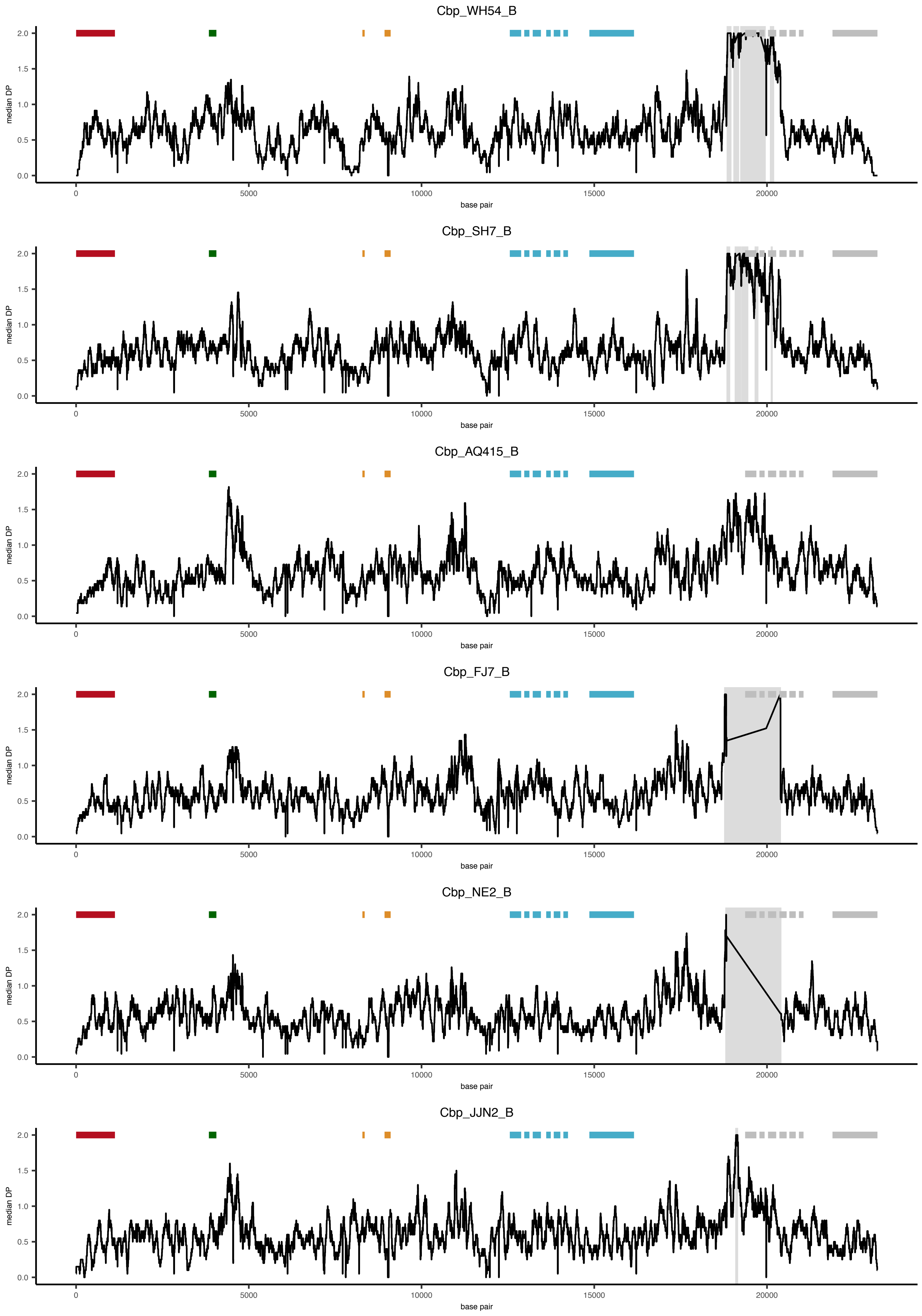
Cbp_WH54_B
Cbp_SH7_B
Cbp_AQ415_B
Cbp_FJ7_B
Cbp_NE2_B
Cbp_JJN2_B
median DP
0.0
0.5
1.0
1.5
2.0
base pair
0
5000
10000
15000
20000

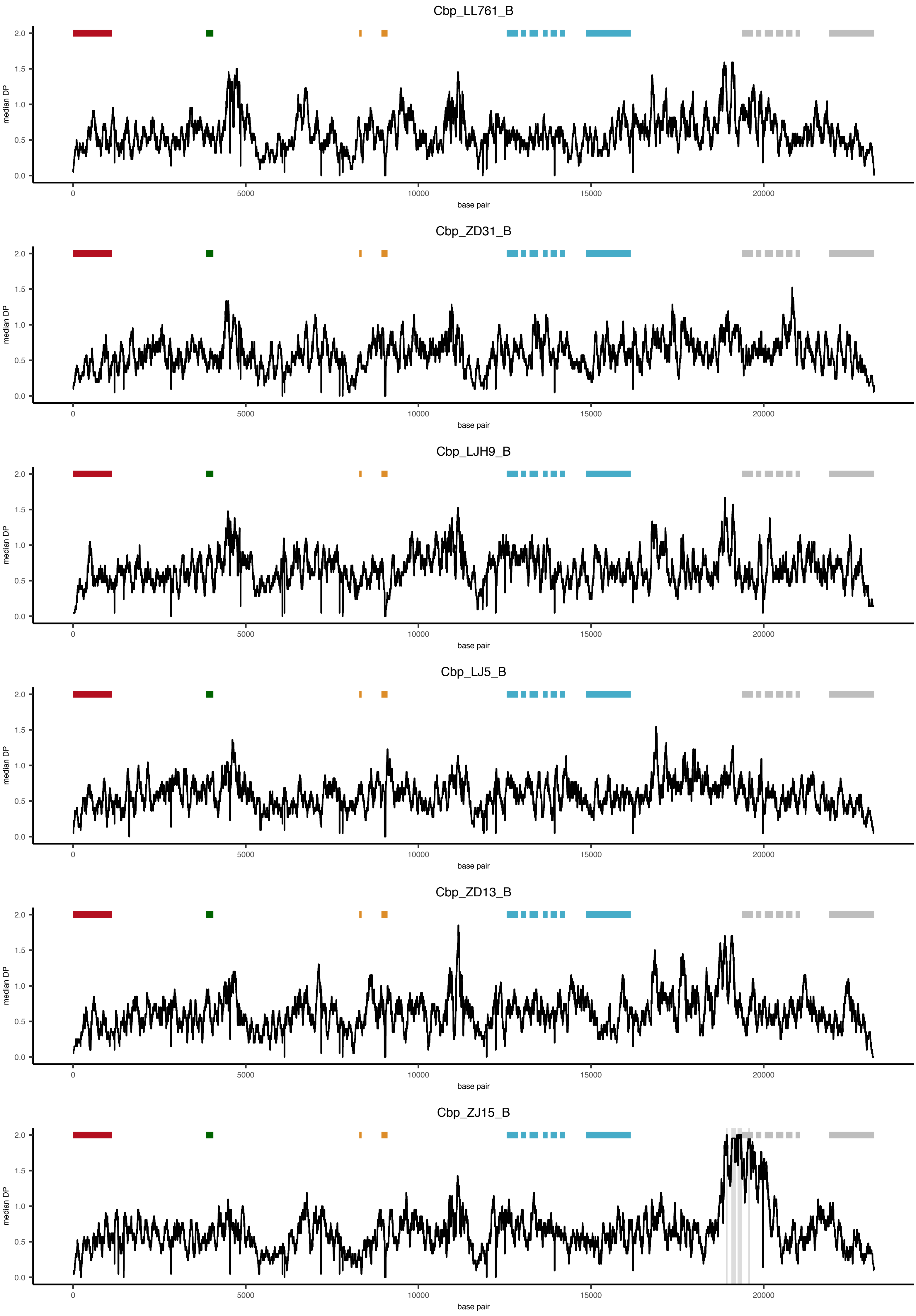
Cbp_LL761_B
Cbp_ZD31_B
Cbp_LJH9_B
Cbp_LJ5_B
Cbp_ZD13_B
Cbp_ZJ15_B
median DP
base pair
0.0
0.5
1.0
1.5
2.0
0
5000
10000
15000
20000

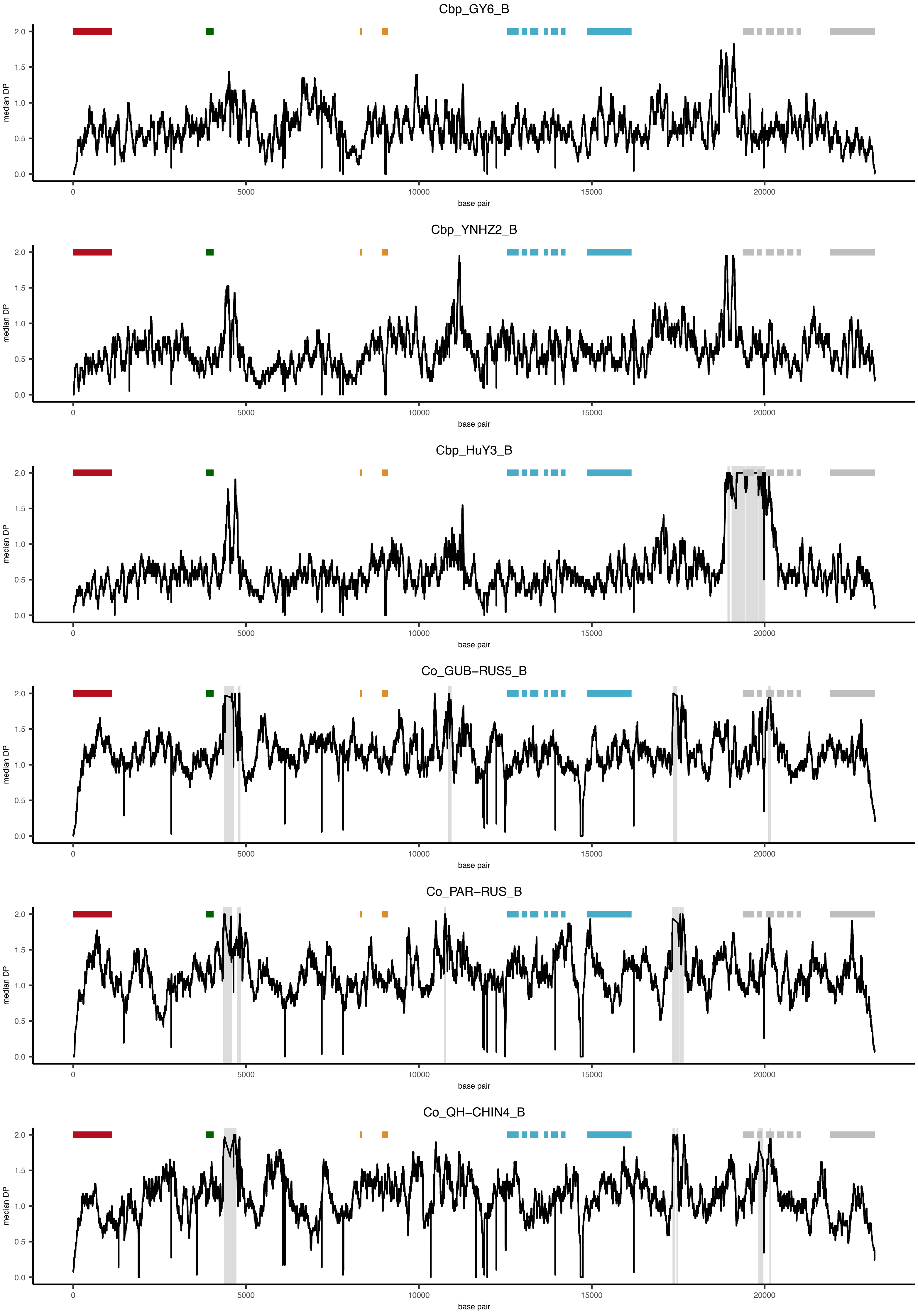
Cbp_GY6_B
Cbp_YNHZ2_B
Cbp_HuY3_B
Co_GUB-RUS5_B
Co_PAR-RUS_B
Co_QH-CHIN4_B
median DP
base pair
0.0
0.5
1.0
1.5
2.0
0
5000
10000
15000
20000

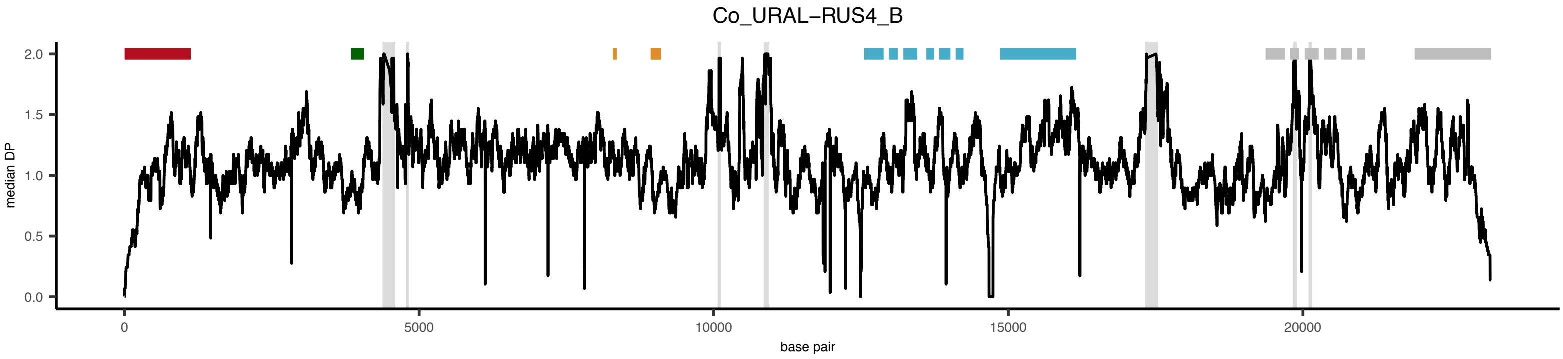

Co_URAL−RUS4_B
median DP
0.0
0.5
1.0
1.5
2.0
0
5000
10000
15000
20000
base pair

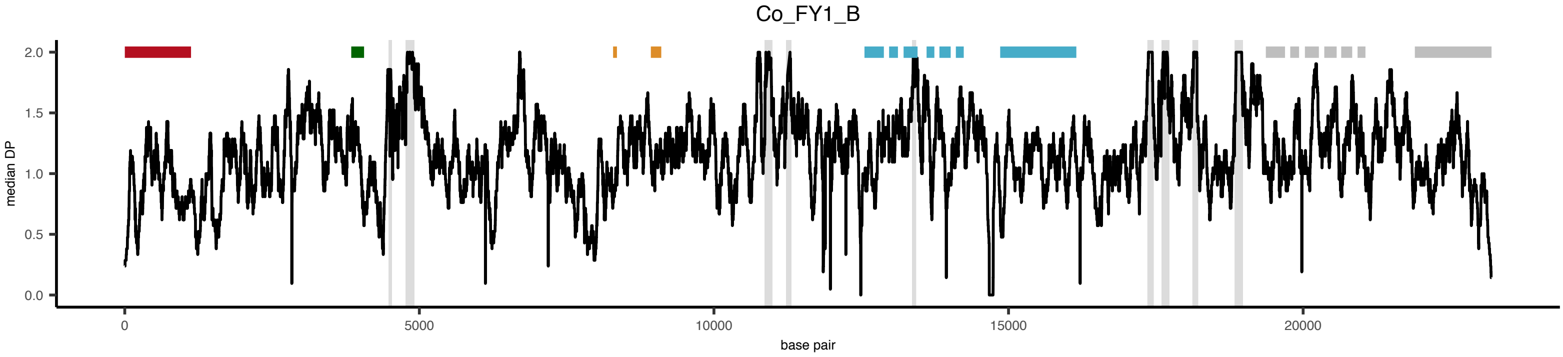

Co_FY1_B
median DP
0.0
0.5
1.0
1.5
2.0
0
5000
10000
15000
20000
base pair

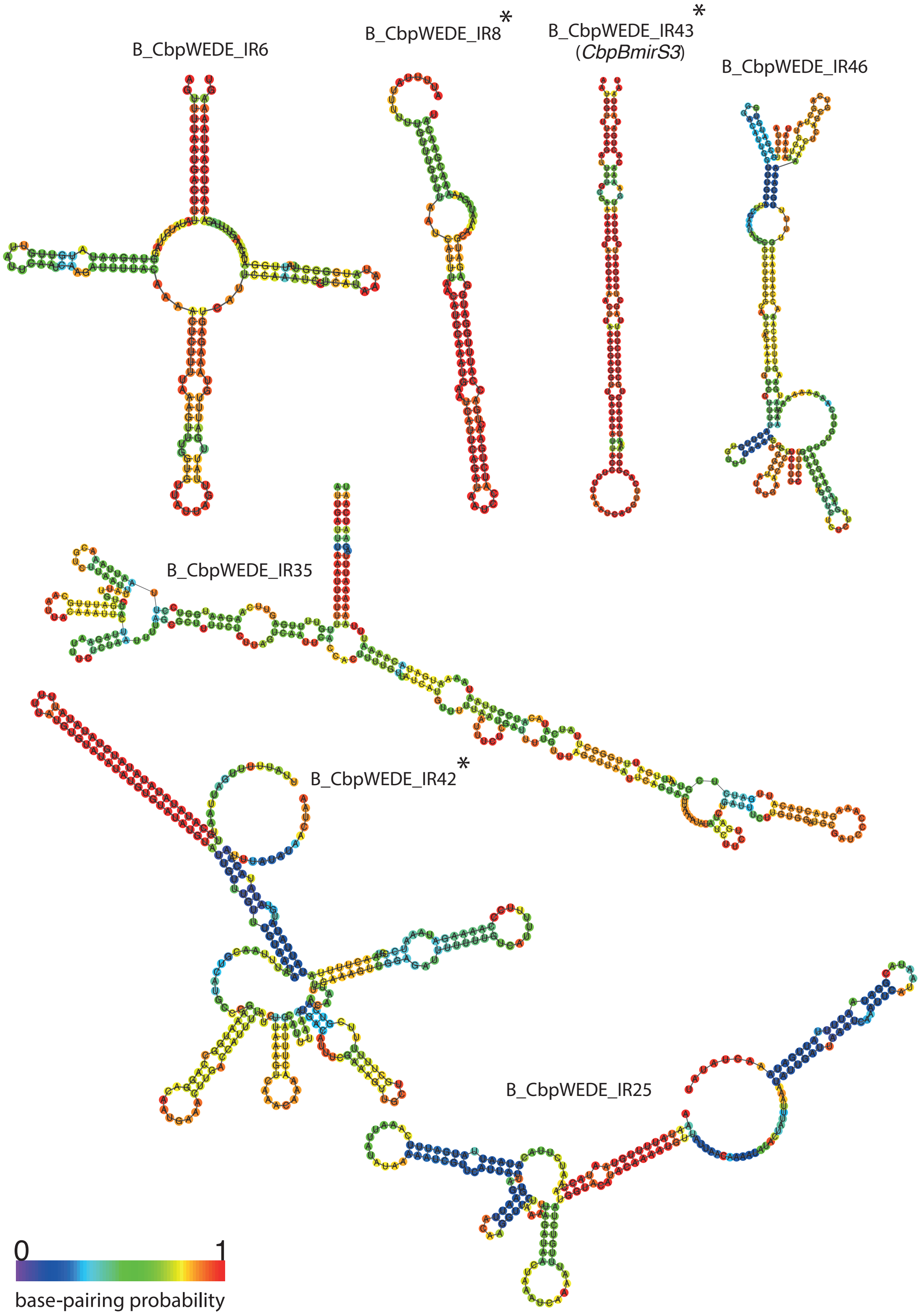

Figure S3. Potential sRNA precursors in *C. bursa-pastoris* subgenome B, folding structure predicted with *rnafold*. The structures are colored by base-pairing probabilities. For unpaired regions the color denotes the probability of being unpaired. Asterisk marks hairpins that passed criteria for sRNA precursors.

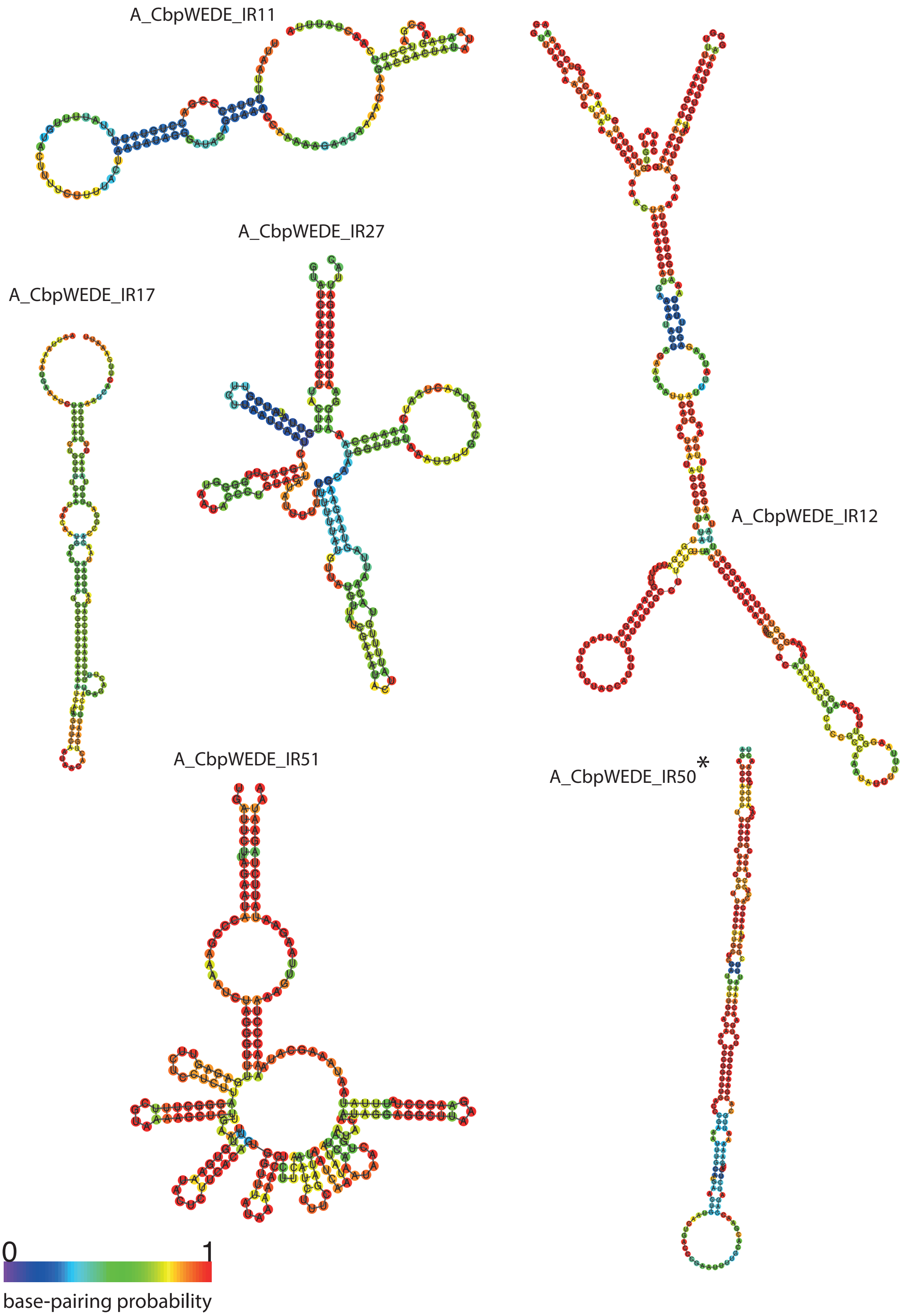

Figure S4 A. Potential sRNA precursors in *C. bursa-pastoris* subgenome A, folding structure predicted with *rnafold*. The structures are colored by base-pairing probabilities. For unpaired regions the color denotes the probability of being unpaired. Asterisk marks hairpins that passed criteria for sRNA precursors.

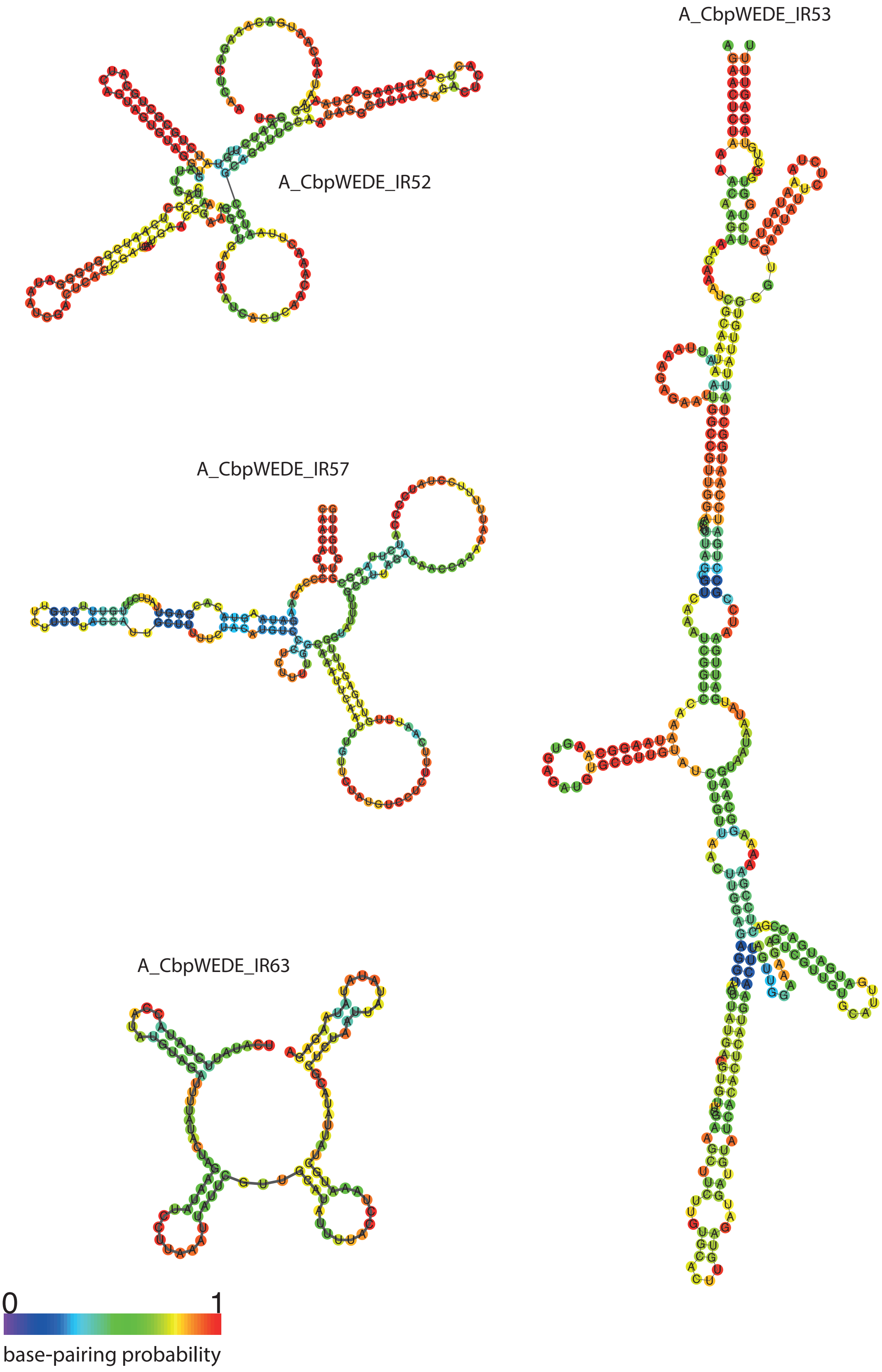

Figure S4 B. Potential sRNA precursors in *C. bursa-pastoris* subgenome A, folding structure predicted with *rnafold*. The structures are colored by base-pairing probabilities. For unpaired regions the color denotes the probability of being unpaired.

References

Huang, H.-R., Liu, J.-J., Xu, Y., Lascoux, M., Ge, X.-J., and S. I. Wright. 2018. Homeologue-specific expression divergence in the recently formed tetraploid *Capsella bursa-pastoris* (Brassicaceae). New Phytol 220:624–635.

Kryvokhyzha, D., Milesi, P., Duan, T., Orsucci, M., Wright, S.I., Glémin, S., and M. Lascoux. 2019. Towards the new normal: Transcriptomic convergence and genomic legacy of the two subgenomes of an allopolyploid weed (*Capsella bursa-pastoris*). PLoS Genet 15: e1008131.

Slotte. T., H.-R. Huang., K. Holm., A. Ceplitis., K. St Onge, J. Chen, U. Lagercrantz, M. Lascoux. 2009 Splicing variation at a *FLOWERING LOCUS C* homeolog is associated with flowering time variation in the tetraploid *Capsella bursa-pastoris*. Genetics **183**: 337–345.
